# Mesenchymal stem cell-derived liver-on-a-chip reconstitutes human liver structure and function

**DOI:** 10.1016/j.crmeth.2026.101545

**Published:** 2026-08-10

**Authors:** Anastasia Brooks, Haotian Yang, Deborah S. Barkauskas, Supun Ranaweera, Da Zou, Sally Goh, Qi Ruan, Kim Bridle, Darrell H.G. Crawford, Xin Liu, Michael S. Roberts, Michael R. Doran, Xiaowen Liang, Chun-Xia Zhao, Haolu Wang

**Affiliations:** 1 Frazer Institute, Faculty of Health, Medicine and Behavioural Science, The University of Queensland, Brisbane, QLD 4102, Australia; 2 Gallipoli Medical Research, Greenslopes Private Hospital, Brisbane, QLD 4120, Australia; 3 Faculty of Health, Medicine and Behavioural Science, The University of Queensland, Brisbane, QLD 4006, Australia; 4 School of Chemical Engineering and Advanced Materials, The University of Adelaide, Adelaide, SA 5005, Australia; 5 School of Biomedical Science, Faculty of Health, Queensland University of Technology (QUT), Brisbane, QLD 4000, Australia

**Keywords:** liver-on-a-chip, microfluidic, human mesenchymal stem cell, cholangiocyte, hepatocyte, endothelial cell

## Abstract

An *in vitro* model of human liver with functional integrity would advance liver research and enable personalized therapeutics. A microfluidic liver-on-a-chip can generate a functional hepatobiliary network using human liver cells and recapitulate blood circulation and bile excretion. However, many current microfluidic liver models lack a biliary component and cannot fully recapitulate the excretory function of the human liver. Here, we develop a microfluidic liver-on-a-chip incorporating functional endothelial cells, hepatocytes, and cholangiocytes derived from a single source of human mesenchymal stem cells. This stem cell-derived liver-on-a-chip reconstructs a simplified liver unit, including a sinusoid, a hepatocyte compartment, and a bile duct supported by barrier integrity and fluid flow. It reproduces integrated organ-level responses to xenobiotics, including hepatic uptake and biliary excretion, and models ischemic liver injury with therapeutic cell recruitment. This technology expands personalized organ-level modeling and holds strong potential for studying tissue development and advancing targeted therapies for liver injury.

## Highlights

• hMSCs are differentiated into hepatocytes, endothelial cells, and cholangiocytes
• A three-channel liver-on-a-chip reconstructs sinusoid, hepatocyte, and bile duct units
• The chip reproduces key liver functions, including hepatic uptake and biliary excretion
• It models ischemic liver injury and recruits therapeutic cells for targeted therapies

## Motivation

Many current microfluidic liver models lack a biliary component and, therefore, cannot fully recapitulate the excretory function of the human liver due to the limited availability of primary human liver cells and proper design of liver-on-a-chip platforms. To address this limitation, we developed a two-stage and feeder-free differentiation approach to generate cholangiocyte-like cells from human mesenchymal stem cells (hMSCs). Furthermore, we engineered a microfluidic liver-on-a-chip system incorporating functional endothelial cells, hepatocytes, and cholangiocytes derived from a single hMSC source, enabling the reproduction of key integrated liver functions.

## Graphical abstract

**Figure:**
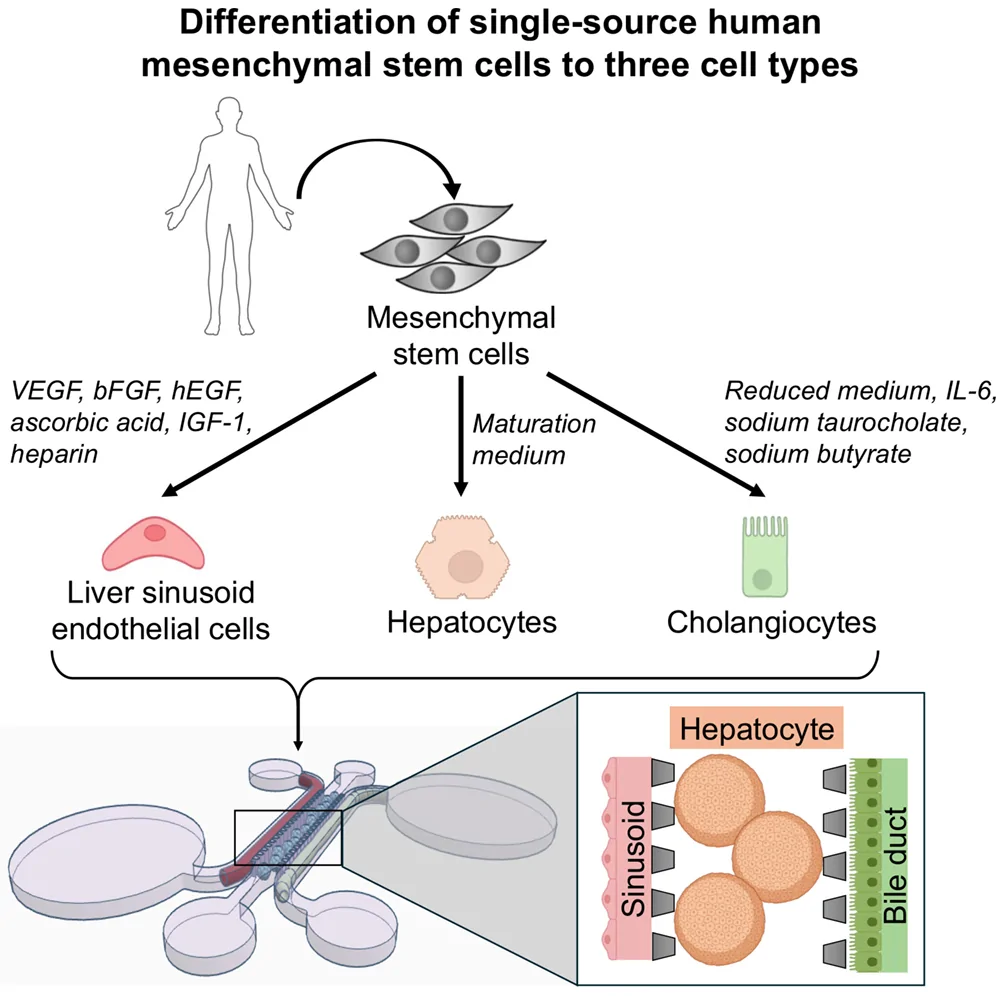

## Teaser

Brooks et al. report a human MSC-derived liver-on-a-chip integrating endothelial cells, hepatocytes, and cholangiocytes from a single donor source. The platform reconstructs the sinusoidal, hepatic, and biliary compartments, supports hepatic uptake and biliary excretion, and models ischemic liver injury with therapeutic cell recruitment for personalized therapy and liver research.

## Introduction

The liver is the largest intracorporeal organ in the human body and plays a critical role in the metabolism and excretion of xenobiotics.^1^ It possesses a unique microenvironment with complex and multiple cell-cell interactions and internal vascular networks.^2^ The human liver is composed of parenchymal cells (hepatocytes, which play a central role in metabolism, detoxification, and bile acid synthesis) and non-parenchymal cells (liver sinusoidal endothelial cells, cholangiocytes, hepatic stellate cells, and Kupffer cells). In a functional liver microstructure, liver sinusoidal endothelial cells serve as a permeable barrier and have fenestrae involved in exchanges between hepatocytes and blood.^3^ Hepatocytes are connected to the intrahepatic bile ducts lined with cholangiocytes for bile excretion.^4^ Neither animal models nor *in vitro* cultures of primary or immortalized human liver cells alone can effectively mimic the metabolism and excretory functions of the liver observed in humans. Thus, there is a pressing need for a model with human liver integrity that could be used to advance fundamental research and develop new and personalized therapeutics.

To reproduce human liver structure and function, all three cell types are needed to maintain the liver integrity for metabolism and excretion. Primary hepatic cells are gold standard sources to model organ level function of the liver and disease pathogenesis.^5^ However, primary human hepatic and biliary cells have been challenging to engineer due to the complexity of their transcription and function and the inaccessibility to liver tissues.^6^^,^^7^ Recently, cells with cholangiocyte characteristics have been generated from human induced pluripotent stem cells (iPSCs).^8^^,^^9^^,^^10^ However, the clinical application of iPSCs raises concerns about the uncontrolled tumorigenic expansion.^11^ Human mesenchymal stem cells (hMSCs) are self-renewing adult stem cells of mesodermal origin that have potential to differentiate into a variety of cell types.^12^ Compared with iPSCs, MSCs are more commonly used in the clinic due to their non-tumorigenesis and non-transgenic phenotype reprogramming.^13^ They represent a promising source for the bioengineering of hepatic cells from a single donor owing to their accessibility, minimal ethical constraints, and ability to be rapidly expanded to clinically relevant quantities.^13^ Advances in MSC technology have enabled the differentiation of hMSCs into endothelial cells and hepatocytes^14^^,^^15^ that exhibit many characteristics of the human liver, including endothelial endocytosis and fenestration, as well as metabolism, detoxification, and bile acid synthesis. A few recent studies have also reported the possibility to generate cytokeratin 19 (CK19)-positive cholangiocyte-like cells from MSCs.^16^^,^^17^ However, there is a lack of a well-established method to efficiently differentiate MSCs into functional cholangiocytes with appropriate characterization.

A microfluidic platform offers the potential to recapitulate the structure and function of hepatic compartments in a controllable dynamic environment to better mimic the *in vivo* condition.^18^^,^^19^ To date, several microfluidic liver-on-a-chip models have been produced, but most failed to recapitulate the excretory function of the human liver due to the lack of a biliary component^20^^,^^21^^,^^22^^,^^23^^,^^24^^,^^25^^,^^26^ and primary human cells.^27^ A simplified liver-on-a-chip +ed by seeding of human umbilical vein endothelial cell line HUVEC and human hepatic progenitor cell line HepaRG.^18^ Although HepaRG cells possess high hepatic functions, their characteristics are still different from those of human liver cells. Only recently, a microfluidic bile duct-on-a-chip containing mouse cholangiocytes cultured under continuous flow was developed to study the bile duct pathophysiology.^28^ In addition, a human vascularized bile duct-on-a-chip was established using organoids derived from primary human bile duct tissue and vascular endothelial cells.^19^ These models exhibited some features such as a simplified bile duct in structure and function and a multicellular platform to study liver diseases. Integration of these features into the hMSC-derived liver-on-a-chip containing multiple functional compartments with fluidic control will make a real difference in liver model development for individual patients.

Here, we report an enhanced human liver-on-a-chip model using functional endothelial cells, hepatocytes, and cholangiocytes differentiated from a single source of hMSCs. It has a hierarchical architecture that captures essential features of a simplified liver unit, including a sinusoid, a hepatocyte compartment, and a bile duct supported by barrier function and fluid flow. This chip exhibits physiologically relevant levels of human liver function for at least 48 h *in vitro*, including sinusoidal fluid flow, hepatic uptake, biliary permeability, and excretion with functional transporters. It also recapitulates liver ischemia injury *in vitro* with the ability of recruiting therapeutic cells. This human liver-on-a-chip offers an effective approach to study liver function and to develop and monitor targeted therapies for liver injury *in vitro*.

## Results

### Differentiation of hMSCs into hepatic and biliary cells

In this study, we chose primary hMSCs as the sole cell source to generate principal cell types of the liver. Before differentiation, we examined the pluripotency of hMSCs at day 0 by immunofluorescence and found that more than 90% of hMSCs highly expressed CD44 and vimentin (Figure S1). Differentiation of hMSCs into hepatocytes (hMSC-HCs) or endothelial cells (hMSC-ECs) was performed according to published protocols.^14^^,^^29^^,^^30^ hMSC-HCs exhibited a hexagonal morphology and significantly increased expression of the hepatocyte-specific marker (*CYP3A4*), which was comparable to the expression in immortalized human hepatocyte (IHH), a human hepatocyte cell line (Figures S2A and S2B). The detailed protein expression patterns, functional properties, and bile canaliculi structures of MSC-derived hepatocytes generated using this protocol have been extensively characterized in prior studies.^14^^,^^29^^,^^30^ Here, we confirmed the expression of a key hepatocyte marker, albumin (ALB), in hMSC-HCs (Figures S2C and S2D). hMSC-ECs highly expressed endothelial markers, including *VWF*, *CD144*, and *VEGFR* (Figure S3).

Our strategy to induce differentiation of hMSCs into the cholangiocyte lineage was based on previously published protocols, with some modifications using feeder-free and defined culture conditions in two stages (Figure 1A).^8^^,^^9^^,^^14^^,^^31^ For stage 1, we focused on the generation of endoderm-like cells. Hepatic endoderm cells, known as hepatoblasts, are bipotential cells in the liver and can be differentiated into both hepatocytes and cholangiocytes *in vitro*. hMSCs were treated with hepatocyte growth factor (HGF), fibroblast growth factor (FGF), and nicotinamide based on previously established hepatic MSC differentiation protocols.^14^ After partial hepatectomy, serum HGF increased significantly,^14^ and HGF and FGF were also detected in endodermal specification during embryonic development, suggesting that those growth factors play important roles in hepatogenesis. Nicotinamide was used to promote stem cell survival and induce hepatic cell regeneration.^32^ By day 9, these cells showed a significant upregulation of genes associated with endoderm (*GATA4* and *FOXA2*), along with the downregulation of genes associated with MSCs.

**Figure 1:**
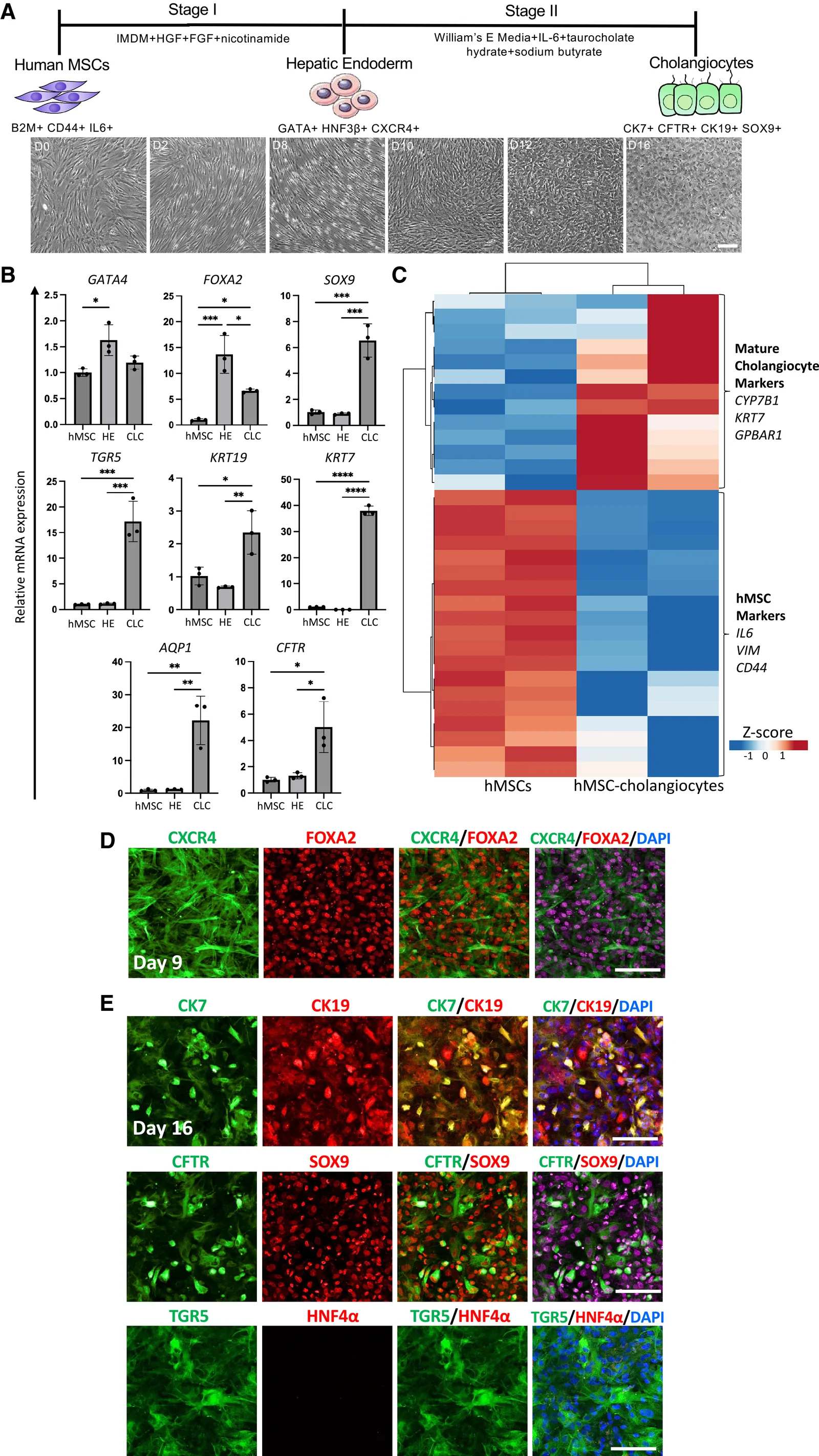
Generation of functional human cholangiocyte-like cells from hMSCs (A) Schematic overview of the timeline (top) and the sequential morphological changes (day 0–16, below) during the two-stage differentiation of hMSCs into cholangiocytes. IMDM, Iscove’s modified Dulbecco’s media; HGF, hepatocyte growth factor; FGF, human fibroblast growth factor-basic; IL-6, interleukin 6. Scale bars, 200 μm. (B) Gene expression profile of hMSC-derived cells at key stages of biliary differentiation as compared with hMSCs, which were used as a negative control. Values are presented as fold change relative to the expression in day 0 hMSCs, set as 1 (*n* = 3). Error bars indicate ± SD. Significance was determined using one-way ordinary ANOVA and Tukey’s post hoc test. ∗*p* < 0.05; ∗∗*p* < 0.01; ∗∗∗*p* < 0.001; ∗∗∗∗*p* < 0.0001. (C) Heatmap of DEG expression in comparing the transcriptomes of hMSCs and hMSC-cholangiocytes. Rows are centered, and unit variance scaling is applied to the rows. Both rows and columns are clustered using the correlation distance and average clustering method. HE, hepatic endoderm; CLC, cholangiocyte. (D and E) Immunofluorescence analyses confirming the high expression of endoderm markers (CXCR4 and FOXA2) on day 9 (D) and biliary markers (CK7, CK19, SOX9, TGR5, and CFTR), not hepatic marker (HNF4α) on day 16 (E) on hMSC-derived cells. Scale bars, 100 μm. See also Figures S1, S2, S3, S4, and S5.

For stage 2, differentiation of these hepatic endoderm-like cells into cholangiocytes, we screened published protocols of hepatic endoderm cell differentiation and tested factors reported to control biliary specification.^8^^,^^9^^,^^31^ Although activin and retinoic acid were reported to induce the expression of early biliary specification markers including *SOX9*, *HNF1B*, and *CK19* in iPSC-derived hepatoblasts *in vitro*, mature biliary markers such as *SCR*, *AQP1*, and *AE2* were less expressed in those differentiated cells.^9^ Thus, activin and retinoic acid were not tested in our stage 2 differentiation protocol. We included interleukin 6 (IL-6), which is present in fetal liver, and sodium taurocholate hydrate, which could not only stimulate differentiation and proliferation of cholangiocytes but also displays antiapoptotic activity on rat cholangiocytes.^31^ To prevent the differentiation of endoderm cells into hepatocytes, sodium butyrate was added after 2 days of sodium taurocholate hydrate treatment.^31^ We also tested the effect of published biliary differentiation medium (BDM) on the differentiation of MSC-derived hepatic endodermal cells (Figure S4), which included linoleic acid-albumin, 3,3′,5-triiodo-L-thyronine, insulin, vitamin C, apo-transferrin, and sodium pyruvate, as well as human growth hormone, epidermal growth factor (EGF), IL-6, and sodium taurocholate hydrate.^31^ As shown in Figure 1A, our final stage 2 differentiation strategy included IL-6, sodium taurocholate hydrate, and sodium butyrate. Cells progressively acquired a cuboidal morphology from day 10 to day 12. By day 16, the majority of cells had undergone drastic morphologic changes (Figure 1A) and also showed a significant increase in the expression levels of cholangiocyte markers, including *SOX9*, *TGR5*, *KRT19*, *KRT7*, *AQP1*, and *CFTR* (Figure 1B).

Transcriptomic analysis of hMSCs and hMSC-derived cholangiocytes using Euclidean hierarchical clustering revealed that hMSC-cholangiocytes are distinct from hMSCs (Figure 1C). 19 of the MSC-related genes, such as *IL6*, *VIM*, and *CD44*, were identified to be expressed only in MSCs and were significantly reduced in hMSC-cholangiocytes. The other 13 cholangiocyte-related genes, such as *KRT7*, *CYP7B1*, and *GPBAR1*, which are specific to biliary cells and responsible for the biliary function, expressed uniquely in hMSC-cholangiocytes but not in undifferentiated hMSCs.^33^ Key genes were validated with RT-qPCR analysis (Figure S5A). Immunofluorescence staining further confirmed that the differentiated cells highly expressed the hepatic endoderm markers CXCR4 and FOXA2 at day 9 (Figure 1D), and hMSC-derived cholangiocytes highly expressed cholangiocyte markers but were negative for the hepatic marker HNF4α at day 16 (Figure 1E), confirming that these cells were terminally differentiated into cholangiocytes using this 16-day differentiation protocol. In addition, to evaluate the canalicular transporters in hMSC-cholangiocytes,multi-drug resistance protein 2 (MRP2)-mediated efflux in hMSC-cholangiocytes was assessed using 5(6)-carboxy-2′,7′-dichlorofluorescein diacetate (CDFDA). As shown in Figure S5B, CDFDA was hydrolyzed intracellularly to an MRP2 substrate, 5(6)-carboxy-2′,7′-dichlorofluorescein (CDF), which was excreted via MRP2 within 20 min of CDFDA removal from the medium. This efflux was inhibited by the MRP2 inhibitor MK571, as indicated by the accumulation of CDF fluorescence in the cells, confirming a functional MRP2 activity in hMSC-cholangiocytes.

### Generation of functional hMSC-cholangiocyte organoids

To discern appropriate conditions for the maintenance, propagation, and functionality of hMSC-derived cholangiocytes, we assessed the potential of hMSC-cholangiocytes to form organoids, which could resemble the elementary units of the biliary system. hMSC-cholangiocytes were cultured in 33% Matrigel, using a three-dimensional (3D) cultural system. After 4 days of culture, hMSC-cholangiocytes formed 3D structures that displayed either a cyst morphology, a tubular morphology, or a mixture of both. The formed hMSC-cholangiocyte organoids highly expressed biliary markers, including CK19, CK7, and SOX9 (Figure 2A), as well as the canalicular transporter BSEP (Figure S5C), indicating that hMSC-cholangiocytes maintained the biliary phenotypes. As we observed the appearance of some pseudoluminal biliary structures, we next used immunostaining for α-tubulin to visualize primary cilia (Figure 2A), which are essential for sensing bile flow and mediating the growth factor signaling pathways.^34^ The organoid polarity was further verified by basolateral and apical localization of β-catenin and CFTR,^31^ respectively (Figure 2A). Approximately ten 3D structures developed per 10^5^ hMSC-cholangiocytes, and the majority (˃80%) of them displayed a cyst morphology. Around 10% of cysts continued to branch tubular structures after culturing for 5 days (Figure 2B). Of note, hMSC-cholangiocyte organoids could be efficiently generated in reduced medium culturing conditions compared with other methods, with uniform and consistent round shape (Figure S5D).

**Figure 2:**
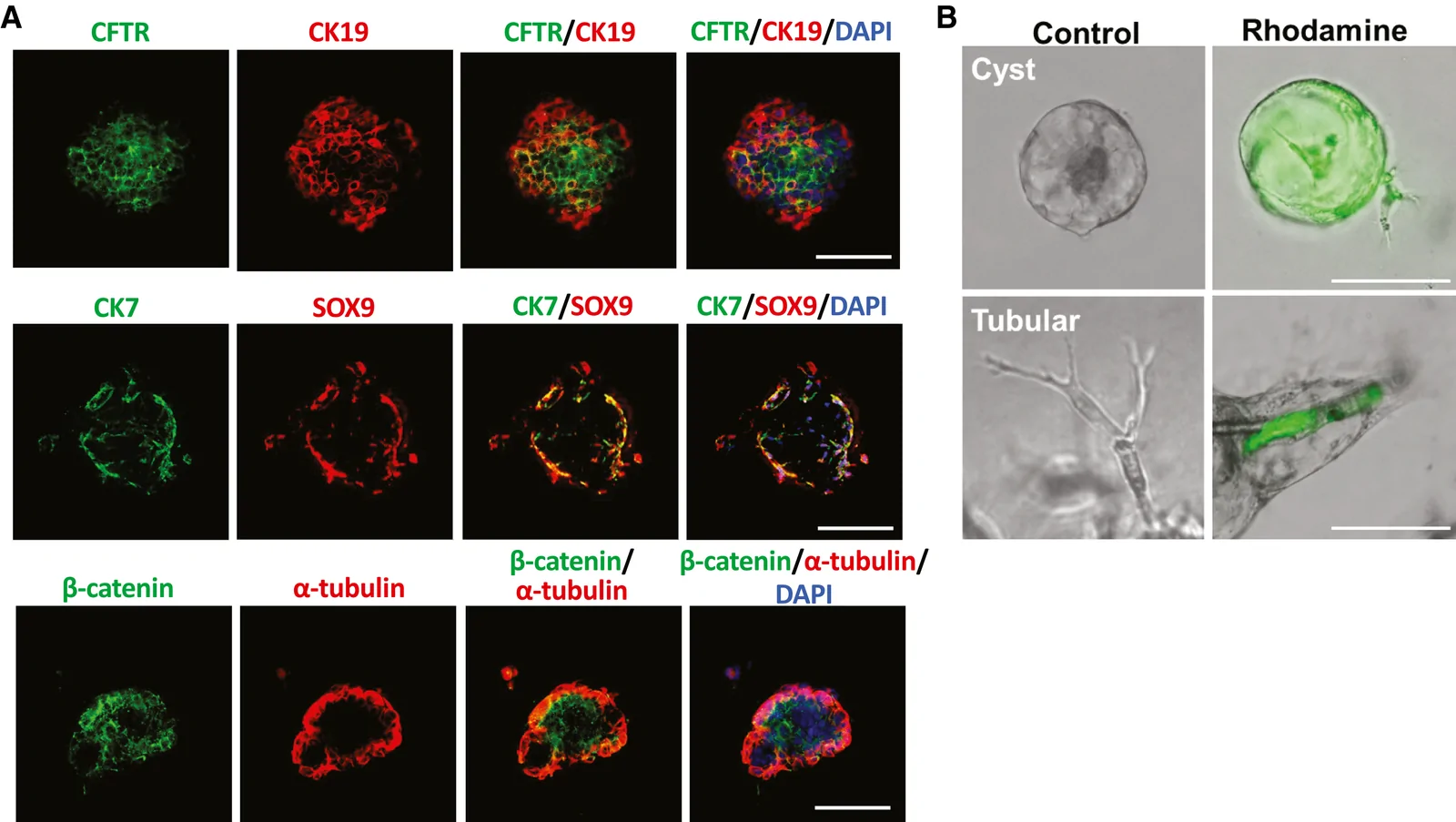
Characterization and functionality of hMSC-cholangiocyte organoids (A) Immunofluorescence staining demonstrating the expression of biliary markers (CFTR, CK19, CK9, and SOX9) and adherens junction markers (β-catenin and α-tubulin) in hMSC-cholangiocyte organoids. Scale bars, 100 μm. (B) Cholangiocyte organoids show cyst and tubular structures (left). The P-gp fluorescent substrate RHD123 was detected in the lumen of cholangiocyte organoids, confirming P-gp functionality (right). Scale bars, 100 μm (top) and 50 μm (bottom). See also Figure S5.

One important function of the human bile duct is the secretion of small substances by various transmembrane channel proteins such as permeability glycoprotein (P-gp), which plays a key role in drug absorption and excretion.^31^^,^^35^ The secretory potential of hMSC-cholangiocyte organoids was confirmed using fluorescent rhodamine 123 (RHD123), a substrate for P-gp. We found that RHD123 was actively secreted to the luminal space of the cyst and tubular structures, evidenced by the detection and accumulation of RHD123 fluorescence in the central lumen of hMSC-cholangiocyte organoids (Figure 2B). This result confirmed P-gp-dependent transfer of RHD123 in MSC-derived cholangiocyte organoids. Taken together, these results suggest that hMSC-cholangiocytes can be used to generate bile duct-like organoids, which display mature secretory functions similar to the native biliary epithelium.

### Fabrication of the liver-on-a-chip

Hepatocytes, which constitute the majority (∼80%) of the hepatic cells, carry out the basic function of the liver such as metabolism and blood detoxification.^1^^,^^36^^,^^37^ Bile acids and salts from the blood are transported into the bile canaliculi and excreted from the liver into the biliary system.^37^ These functions are highly sensitive to multiple factors in the local tissue microenvironments including cell-cell interactions such as hepatocytes and cholangiocytes. Conventional 3D organoids typically consist of primary cells such as hepatocytes and mimic the organ function but lack a dynamic microenvironment. As a result, conventional 3D *in vitro* models and tissue culture methods often fail to reproduce the structural and functional characteristics of the liver. Consequently, system-level studies of liver biology and pathology largely rely on animal models.^38^ However, animal studies cannot recapitulate the human physiological environments. In this context, a microfluidic organ-on-a-chip platform offers advanced cell-cell interactions^18^ and cell-extracellular matrix junctions in a controllable dynamic media flow, allowing high-resolution imaging and metabolic and biochemical assays in real time.^39^^,^^40^ The dynamic aspect of the 3D organoids is ideal for the recapitulation of blood circulation and bile excretion in the liver. A few liver-on-a-chip systems have been produced previously, but most of them only have one compartment or have a combination of hepatocytes and non-parenchymal cells, often endothelial cells, but lack counterflow and a biliary component due to the difficulty in accessing primary human cholangiocytes.^18^^,^^20^^,^^21^^,^^22^ We, therefore, examined whether hMSC-derived functional hepatic cells could be used to engineer a functional liver-on-a-chip to analyze human liver function and to mimic and monitor cell therapy for injured livers.

A three-channel multifunctional microfluidic device was designed for recapitulating the structural and functional properties of the liver unit with fluid flow and containing a sinusoid, hepatocyte spheroids, and a bile duct (Figures 3A–3C). The microfluidic device made of polydimethylsiloxane (PDMS) elastomer consists of three independent but interconnected microchannels, showing a basic replication of the liver unit. Culture medium flows through the sinusoid channel to the hepatocyte channel to mimic the “Space of Disse.” Oxygen consumption by cells and medium evaporation were the other major issues. In addition, the effect of various strain conditions on cell behavior in channels was tested by using a pump. However, the pump-induced flow tended to detach cells and destroy the collagen gel. Hence, larger reservoirs (5.25 mm in diameter) at the outlets were incorporated for pumpless flow (Figure S6A), allowing the long-term culturing system. The design of pillars is critical to prevent cell infiltration from the other channels during cell pipetting (Figures S6B and S6C). Figure S6D shows an actual liver-on-a-chip device filled with dyes. Hexagonal and octagonal pillars failed to support the tube wall formation, and most cells still infiltrated through the gaps into the central channel (Figures S6B and S6C). Therefore, trapezoidal pillars were selected for better cell retention. The final design of the device (Figures 3B–3D) replicates the hierarchical architecture of the liver unit, housing a sinusoid, a bile duct, and a hepatic compartment in a controllable way. Compared with two-dimensional culturing, primary human hepatocyte spheroids retain morphology, viability, and hepatocyte-specific functions as well as maintain phenotypic stability for longer culture period and functional analysis. The structure of a spheroid possesses a microenvironment, which is similar to the liver with cell-cell interactions.^41^ Therefore, hMSC-derived hepatocyte spheroids were introduced into the central channel of the chip to recapitulate the function of hepatocytes in the liver unit. The outer channels of 300 μm were seeded with endothelial cells or cholangiocytes to recreate an endothelial barrier and mimic the sinusoid or bile duct (Figures 3D and S7A). The barrier formed by endothelial cells was further confirmed in a collagen-based migration assay. MSC migration was induced by the stromal cell-derived factor-1 (SDF-1) gradient, where a significantly higher degree of cell congestion was observed at the collagen barrier in the presence of endothelial cells (Figure S7B). These results demonstrated that the endothelial cells created an effective barrier between channels. The inner channel of 600 μm was seeded with hepatocyte spheroids. The designed microfluidic system used pumpless passive flow to model the dynamic mechanical strain observed in the living liver.

**Figure 3:**
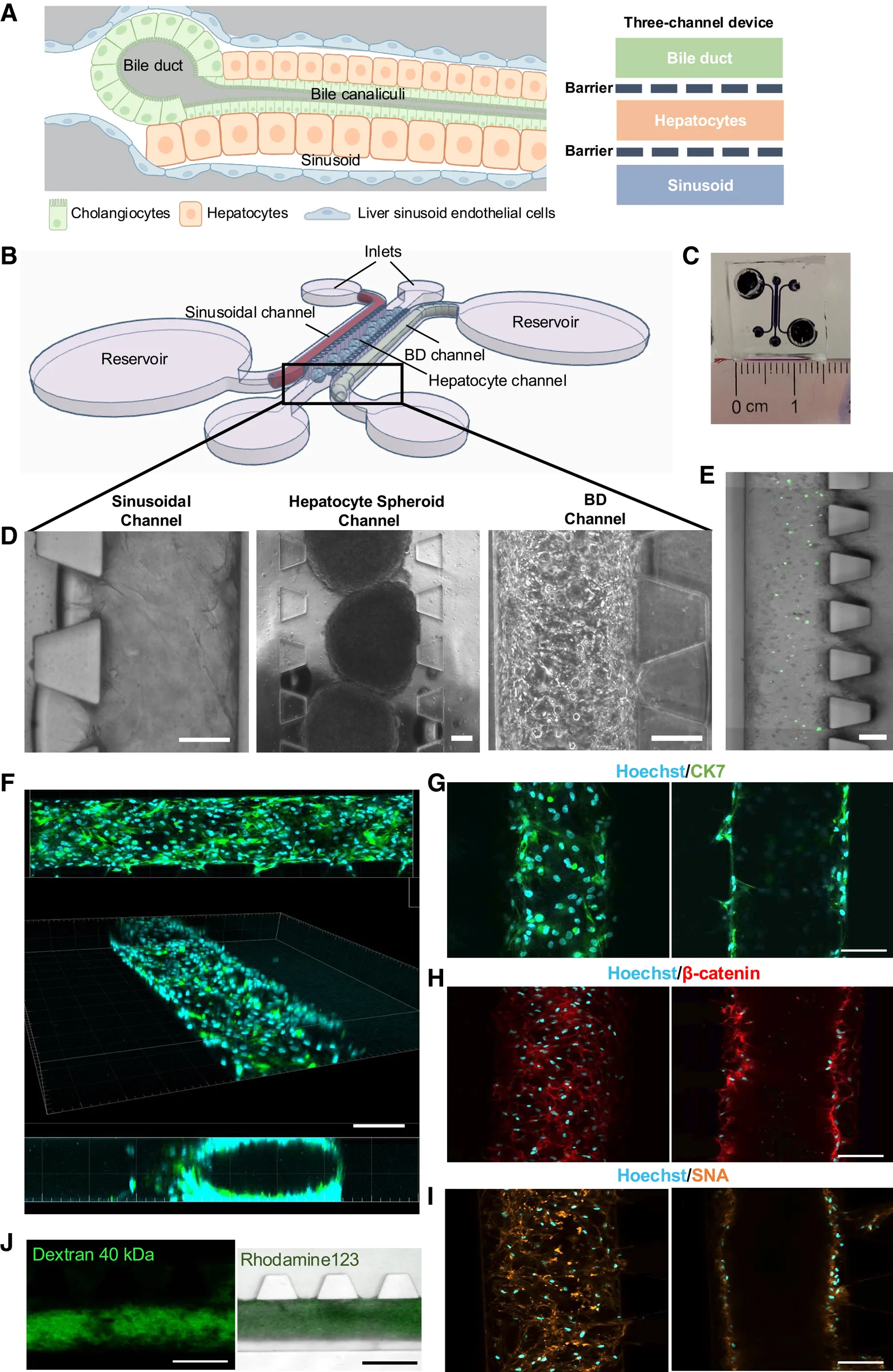
Fabrication and characterization of the hMSC-derived liver-on-a-chip microfluidic device (A) Schematic representative of the liver unit with sinusoids, hepatocytes, and bile ducts. (B) Schematic diagram of a microfluidic liver-on-a-chip device with three microchannels replicating a sinusoid, hepatocyte spheroids, and a bile duct (BD) compartment. (C) Photograph of an actual liver-on-a-chip device filled with dyes. (D) Representative bright-field images of the device containing a sinusoidal compartment, a hepatocyte compartment, and a bile duct compartment, which were made by hMSC-derived endothelial cells, hepatocyte spheroids, and cholangiocytes, respectively. BD, bile duct. Scale bars, 100 μm. (E) Representative image of z-average 100-nm DiI-loaded PET NPs in a channel for 10 min for the calculation of the flow speed within empty and bile duct channel. PET, polyethylene terephthalate. Scale bars, 100 μm. (F) Representative z stacking confocal images with longitudinal (middle) and cross-sectional (bottom) views of hMSC-cholangiocytes in the bile duct compartment, forming a monolayer with the cylindrical channel. Scale bars, 200 μm. (G–I) Immunofluorescence images across the top (left) and middle (right) of the channel stained with CK7 (G), β-catenin (H), and SNA (I). Scale bars, 100 μm. (J) Uptake of 40 kDa FITC-dextran solution (left) and RHD123 (right) into the lumen of the hMSC-cholangiocyte bile duct. Scale bars, 200 μm. See also Figures S6 and S7.

To maximize collagen and cell adhesion, the microfluidic devices were first treated with oxygen plasma to reduce hydrophobicity, followed by 2 h of 0.01% poly-L-lysine (PLL) coating, and dried overnight. Collagen gel with hMSC-derived hepatic spheroids was introduced into the central channel and allowed to set prior to laminin coating. hMSC-derived cholangiocytes were seeded into the outer channel coated with 0.2 mg/mL collagen and 100 μg/mL mouse laminin, which formed an appropriate biliary tube within 24 h. hMSC-ECs were seeded into the other outer channel. Both bile duct and sinusoidal channels formed a confluent and compact epithelial monolayer within 24 h, respectively, while spheroids were fitted and squeezed tightly together to minimize gaps in the hepatic channel (Figure 3D).

Flow velocities of the empty or bile duct compartments were measured by real-time tracking of lipophilic fluorescent stain DiI-loaded polyethylene terephthalate (PET) nanoparticles (NPs) throughout the whole channel (Figure 3E). The average flow rate under pressure gradient was calculated to be 0.04 μL/min in empty channels and 0.006 μL/min in channels seeded with hMSC-cholangiocytes or hMSC-ECs (Figure S7C), both of which are within the range of acceptable flow rates.^42^ Flow velocity was not constant and decreased toward the end of the channel (Figure S7C) due to outflow to the neighboring channels, even in presence of collagen gel. This confirmed that the central channel received fluid from the outer channel (sinusoidal compartment). Direction of flow through the central channel was determined by volume in either of the large reservoirs. More volume in the sinusoidal reservoir would direct flow from the sinusoidal channel through hepatic channel to the biliary to model the *in vivo* condition. Therefore, co-culturing of these hepatic cells in the designed device was under physiological fluid flow, which enabled the formation of organ-level structures that closely resemble the normal tissue-tissue interface of the liver unit.

### Characterization of the liver-on-a-chip

We used immunofluorescence staining to characterize the bile duct compartment in the device formed by hMSC-derived cholangiocytes. Remarkably, CK7 staining showed that hMSC-derived cholangiocytes grew into a compact monolayer, forming a tube with two open ends connected to the large reservoirs (Figure 3F). z stack imaging revealed empty space within the tube, confirming the presence of a lumen (Figure 3F). The hMSC-derived cholangiocytes formed a bile duct compartment, which retained positive expression of the biliary marker CK7 (Figure 3G). The polarity of the bile duct compartment was verified by basolateral and apical localization of β-catenin and Sambucus nigra lectin (SNA), respectively (Figures 3H and 3I). β-catenin was highly expressed at cell-cell junctions, validating the formation of tight junction. SNA is an apical surface marker, which appeared to be correctly apically stained on the pillar side, with the basal surface facing the hepatocyte channel (Figure 3I).

The tight junctions between cells are critical for the selective uptake of molecules for excretion. High permeability of the bile duct leads to the leakage of toxic waste into the local tissues.^43^ To assess whether the hMSC-derived cholangiocyte compartment recapitulated the barrier function of the bile duct, 40 kDa fluorescence isothiocyanate (FITC) dextran was perfused to the lumen, and the fluorescence was assessed after 1 h. The formed bile duct demonstrated low permeability, with no obvious leakage of dextran in the matrix (Figure 3J). A fully super confluent monolayer is required for the minimal permeability. The increase in bile duct permeability is indicative of dysfunction and a variety of liver diseases, including primary biliary cirrhosis and cholangiocarcinoma.^43^ Therefore, it is vital to recreate a bile duct tube to model the accurate *in vivo* conditions of patients. The P-gp transporter function was further assessed in the channel of bile duct by using RHD123. The tube was incubated in 100 μM RHD123 for 5 min and then washed. After 30 min, the confocal imaging showed the significant uptake of RHD123 into the lumen (Figure 3J), indicating that the function of P-gp transporter remained in the bile duct compartment. Together, these observations confirmed that the bile duct compartment formed by hMSC-derived cholangiocytes displays a range of typical physiological biliary functions.

### MSC-derived liver-on-a-chip for modeling hepatic uptake and biliary excretion

We next explored whether this microfluidic device could model the *in vivo* hepatic uptake and biliary excretion. RHD123 is a lipophilic dye, which can be rapidly taken up by hepatocytes from sinusoids and excreted from hepatocytes into the bile subjected to the P-gp transporter. As a consequence, RHD123 was used as a substrate to assess the P-gp function. We first used intravital imaging methods to investigate the *in vivo* hepatic disposition and biliary excretion of RHD123 in the exposed liver of anesthetized rats after intravenous dosing (Figure 4A). RHD123 fluorescence was taken up by hepatocytes from sinusoids and excreted from the hepatocytes to the bile duct. The presence of cyclosporine A (CyA), a P-gp transporter inhibitor, markedly increased the RHD123 fluorescence in the hepatocytes at 150 min after administration, indicating slower biliary excretion of RHD123 compared with RHD123 alone (Figure 4A). Inhibition of P-gp function by CyA pretreatment resulted in the impairment of RHD123 biliary excretion and accumulation of RHD123 in hepatocytes.

**Figure 4:**
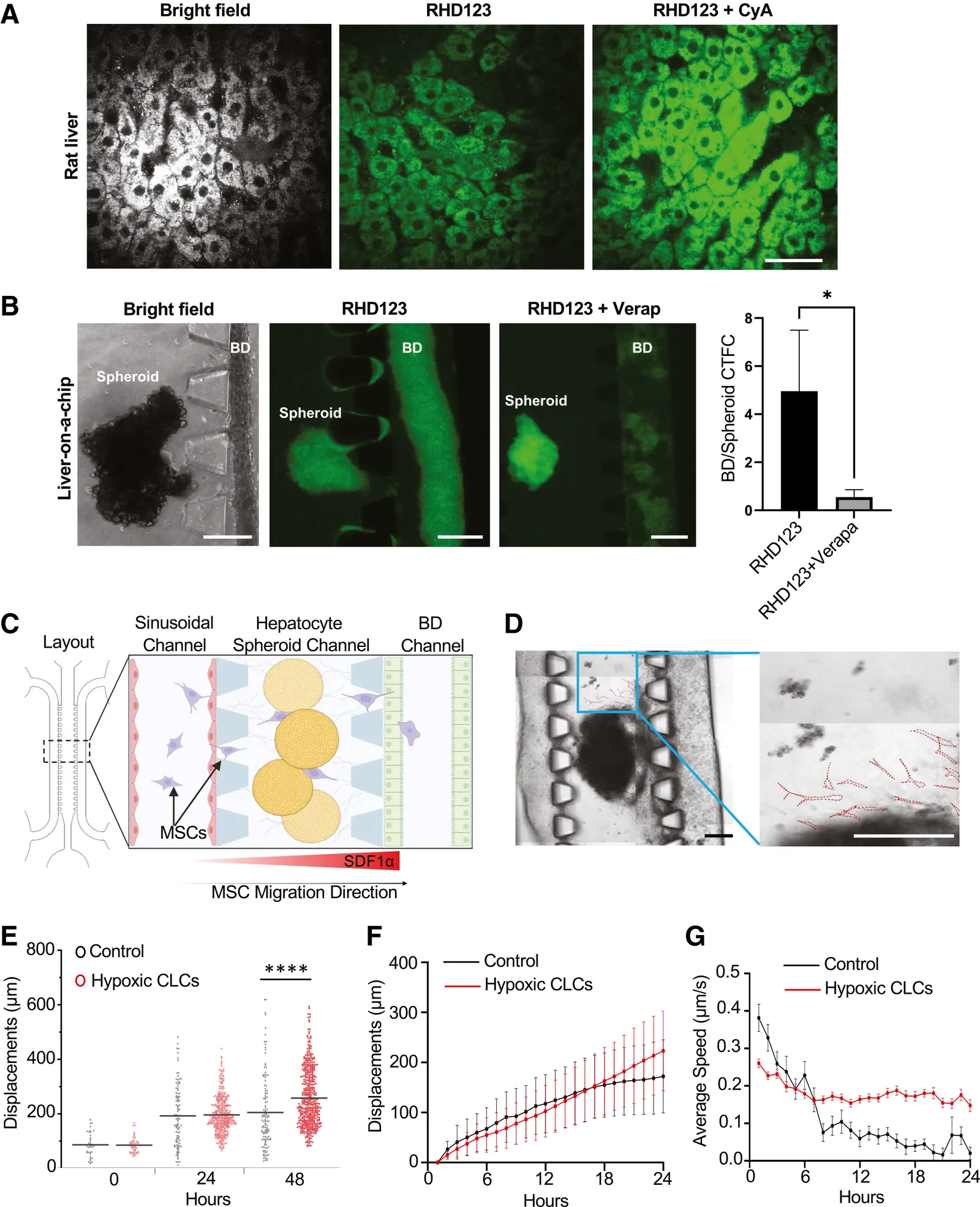
Functional properties of the hMSC-derived liver-on-a-chip (A) Representative images demonstrating the P-gp fluorescent substrate RHD123 was taken up by hepatocytes and secreted from hepatocytes to the bile duct in the rat liver. Images were taken at 150 min after RHD123 or RHD123 and CyA injection in rat liver. RHD123, rhodamine 123; CyA, cyclosporine A. Scale bars, 40 μm. (B) The hMSC-derived liver-on-a-chip recapitulates the liver function where RHD123 was taken up by hepatocyte spheroids and excreted into the bile duct compartment. Liver-on-a-chip was imaged and quantified at 120 min after adding RHD123 or RHD123 and Verap to the chip. Verap, verapamil. BD, bile duct. Scale bars, 200 μm. (C) Schematic of MSC migration from the sinusoidal compartment to the bile duct compartment, where hypoxic hMSC-cholangiocytes secrete SDF-1α and produce a chemotaxis gradient throughout the channel. (D) Representative bright-field image of MSC migration in each compartment, with the enlarged image showing an individual MSC circled by dotted red lines. Scale bars, 200 μm. (E) Distribution of MSC displacement through the sinusoidal and hepatocyte channels toward the bile duct channel in the control (normoxic condition, negative control) and hypoxic conditions. Black lines indicate the mean. Points represent individual cells; *n* = 247 (control) and *n* = 942 (hypoxic condition). CLC, cholangiocyte. (F) Displacement of individual MSCs in negative control (*n* = 37) and hypoxic (*n* = 150) liver-on-a-chip over 24 h. (G) Inferred average speed of cells in the negative control (*n* = 37) and hypoxic (*n* = 150) liver-on-a-chip. Control: normoxic condition of the hMSC-derived cholangiocyte channel; hypoxic CLCs: hypoxic condition of the hMSC-derived cholangiocyte channel. Error bars indicate ± SD. Significance was determined by Student’s *t* test (B and E). ∗*p* < 0.05; ∗∗∗∗*p* < 0.0001. See also Figure S8.

Comparable results were yielded in the developed MSC-derived microfluidic device. The hMSC-derived ECs and cholangiocytes were seeded in the outer channels to form a monolayer sinusoid and bile duct (Figure 3D), while hMSC-derived hepatocyte spheroids were added to the middle channel (Figure 4B). To mimic the *in vivo* condition, we further induced RHD123 in the sinusoidal compartment, and the device was imaged after 120 min. The fluorescence of RHD123 was shown in the spheroids, demonstrating the uptake of RHD123 from the sinusoids to hepatocytes. The intensity of fluorescence was similar in the spheroids at left, middle, and right ends of the channel (Figures S8A and S8B), indicating that the channel length did not have a significant impact on hepatic uptake. RHD123 was further secreted to the luminal space of the bile duct channel. To determine whether the hMSC-derived liver-on-a-chip indeed reflected the activity of a liver unit, the device was incubated with verapamil (a P-gp inhibitor)^44^ before adding RHD123, which significantly prevented the secretion of RHD123 into the bile duct and enhanced the accumulation of RHD123 in the hepatocyte spheroids (Figure 4B). The ratio of the fluorescence intensity between the hepatocyte channel and the bile duct channel confirmed the verapamil significantly inhibited RHD123 excretion into the bile duct. Taken together, the hMSC-derived liver-on-a-chip exhibited *in vivo*-like hepatic transporter and biliary excretion properties of the liver unit.

### MSC-derived liver-on-a-chip mimics cell therapy for the injured liver

As existing *in vitro* models have limited capacity to faithfully mimic both human liver physiology and disease states, we further explored whether this liver-on-a-chip lined with MSC-derived three hepatic cells could model a diseased liver. Ischemic liver injury is a condition defined as a liver injury caused by insufficient blood flow to the liver.^45^ It is not an uncommon condition and accounts for 1%–2.5% of patients admitted to an intensive care unit.^46^ Ischemic liver injury can cause biliary epithelium injury and lead to subsequent bile duct structure, which is widely known as ischemic cholangiopathy. The liver chip was subjected to hypoxic conditions to simulate ischemic liver injury, and then cultured in a hypoxia chamber incubator at 37°C in 3% O_2_, 5% CO_2_, and 92% N_2_ for 4 h. The hypoxic markers, including *HIF1A*, *VEGFA*, and *EDN*, were assessed to confirm the hypoxic condition of the bile duct channel formed by hMSC-derived cholangiocytes (Figure S8C). SDF-1 was reported to be upregulated after hepatic ischemic injury and could be employed as a therapeutic target.^47^ In our study, SDF-1α concentration was quantified in the hypoxic hMSC-cholangiocyte channel, showing an expression of two times higher than that in the normoxic condition (Figure S8D).

Recently, MSC therapy has been suggested as an effective approach for the treatment of ischemic liver injury. MSC properties of homing, regeneration, and immunomodulation have shown high potential for alleviating hypoxia-induced injury in tissues.^48^^,^^49^^,^^50^^,^^51^ Therefore, we monitored the MSC migration toward the hypoxic bile duct compartment in the developed device. Given that the mechanism of MSC homing to the injured tissues is mediated by the SDF-1-CXCR4 axis,^52^^,^^53^ we propose that MSCs seeded in the sinusoidal channel would migrate toward the injured bile duct, where hypoxic cholangiocytes secrete SDF-1α, producing a chemotaxis gradient throughout the channel (Figure 4C). To determine if our device could sustain a chemotaxis gradient, we perfused 4 or 20 kDa dextrans through the collagen gel to simulate the SDF-1α gradient for migration studies (Figure S8E). Based on the fluorescence intensity plots of 4 and 20 kDa (Figures S8F and S8G), SDF-1α (10 kDa) was calculated to take 4.7 h to saturate the collagen gel.

MSCs migrating to the normoxic or hypoxic hMSC-cholangiocytes channels were tracked individually over 48 h, with images taken every hour. The average displacement of MSCs in hypoxic conditions was significantly larger than that in normoxic conditions at 48 h (Figure 4E). The representative bright-field image showed MSCs migrating toward hypoxic hMSC-cholangiocytes, which were tracked individually by dotted red lines (Figure 4D). Randomly selected cells (control: *n* = 37; hypoxia: *n* = 150) were plotted with displacement manually against time to obtain individual cell migration path and speed. The individual cell displacement in normoxic conditions began to plateau after 12 h, while MSCs continued to migrate up to 24 h in hypoxic conditions (Figure 4F). Additionally, MSCs showed rapid motility in the first 6 h for both conditions, which, thereafter, either decreased considerably in normoxic conditions or plateaued in hypoxic conditions (Figure 4G). Thus, hypoxic conditions promoted directional MSC migration compared with normoxic conditions, as indicated by the higher persistence, migration cell number, and a lower directional change rate (Figures S8H and S8I). This device allowed for real-time tracking of therapeutic cell migration in different conditions, which can be potentially used for monitoring and modeling of cell therapy in injured liver tissues.

## Discussion

Taken together, these results demonstrate that the MSC-derived liver-on-a-chip reproduces key structural, mechanical, and functional properties of the human liver unit. It houses a sinusoid, a hepatocyte compartment, and a bile duct, with the functions of uptake and excretion of small molecules such as RHD123, which is one of the key functions of the liver. We were also able to model liver injury and monitor therapeutic cells in the injured liver model. All the liver cells used in this chip are from a single primary cell source, paving the way for potential applications of this technique in personalized organ modeling for individual patients in the future.

The source cell for current *in vitro* human liver models includes liver cell lines, primary liver cells isolated from cadaver donors, and liver cells derived from hMSCs, embryonic stem cells (ESCs), or iPSCs.^18^^,^^20^^,^^21^^,^^22^ However, primary liver cells are almost inaccessible in many clinical settings,^6^ and culturing them remains problematic.^5^ Some hepatic cell lines, such as HepaRG cells, possess high hepatic functions, but their characteristics are different from those of human liver cells. Most studies, to date, have focused on generating hepatocytes from MSCs and have yielded protocols that give rise to mature hepatocytes suitable for *in vitro* modeling of liver diseases and predicting drug toxicology. Recently, cholangiocytes were generated from human ESCs, iPSCs,^8^^,^^9^^,^^10^ and fetal liver cells,^54^ demonstrating that it is possible to derive this lineage from stem cells *in vitro*. Compared with MSCs, ESCs and iPSCs raise concerns due to the possibility of uncontrolled tumorigenic expansion.^11^ iPSCs also display more genetic and epigenetic abnormalities for pharmaceutical studies on reprogramming and transcriptional principles. In this context, MSC-based approaches have less concerns about transgene reactivation and subsequent tumorigenesis that may affect the safe use of stem cell-derived liver cells.

Our study demonstrates an approach to mimic physiological biliary development through a directed differentiation protocol with chemically defined and serum-free conditions that differentiates functional cholangiocyte cells from MSCs that can self-organize into bile duct-like structures. This protocol was designed to generate cholangiocytes *in vitro* with high efficiency that closely resemble primary cholangiocytes at the transcriptional and functional levels for the next step of developing liver-on-a-chip. Cholangiocyte specification with this approach appears to be very efficient, as 80% of the structures that formed in 3D cultures consisted of cholangiocyte-like cells and displayed bile duct characteristics with functional biliary markers. It is also important to assess the maturity of hMSC-derived cholangiocytes in liver-on-a-chip. The typical bile duct polarity and biliary markers and the presence of primary cilia, together with the ability to transport RHD123, demonstrated a mature and functional bile duct derived from MSCs in the developed liver chip. Our protocol will also enable various future applications, including developmental studies, disease modeling, drug screening, and the development of new therapeutics.

To model the functional hepatic unit *in vitro*, hepatocytes must be connected to a bile excretion system and supported by vascular fluid, contributing to the long-term maintenance of the functional liver unit. This has not been possible using conventional cell culture models that are currently used to study liver biology. A microfluidic liver-on-a-chip device is an ideal model to generate a functional hepatobiliary network using human liver cells and recapitulate blood circulation and bile excretion, which is necessary for liver studies. Previously developed liver-on-a-chip models usually contain human hepatocytes and endothelial cells in a dual microchannel, with continuous medium perfusion but without a biliary compartment. They either fail to generate *in vivo*-like barrier functions or have difficulties in mimicking biliary excretion.^18^^,^^20^^,^^21^^,^^22^ Only recently, two microfluidic bile duct-on-a-chip models were developed as a potential tool to study the pathophysiology of the bile duct that contain a functional cholangiocyte compartment and are cultured under continuous flow.^19^^,^^28^ By integrating these features and leveraging the results of hMSC-derived hepatic cells, we engineered the first microfluidic human liver-on-a-chip that permits the application of physiological fluid flow and passive cyclic strain to three compartments of a liver unit separated by the laminin-coated flexible membrane, while simultaneously enabling the analysis of molecular hepatic uptake and excretion across the tissue barrier. This device also replicates hypoxia-induced liver injury as seen in patients with ischemia. The hMSC-derived hepatic cells were cultured in three independent but interconnected microchannels, allowing the accessibility to real-time imaging and monitoring of cell migration at a single-cell level for individual channels, while maintaining appropriate cell and tissue *in vivo*-like functions. Therefore, the therapeutic cell migration was monitored in hypoxic or normoxic conditions to mimic cell therapy in the injured liver tissues. Thus, this designed platform could potentially facilitate future investigations into the molecular mechanisms of liver function in the normal and diseased states and targeted therapies for liver or bile duct diseases of individual patients.

### Limitations of the study

This MSC-derived liver-on-a-chip is not designed to engineer a whole living liver with all of its functionalities. Of note, it is a synthetic platform that attempts to reconstitute essential functions of the liver unit that cannot be modeled currently. In addition, the functional assays in this study were short-term processes that are commonly assessed over minutes to hours in hepatobiliary organ-on-chip systems. Consistent with this, previous studies have evaluated dextran permeability over minutes and transporter-mediated substrate handling over short incubation periods,^19^^,^^28^ demonstrating that these short-term assays can provide physiologically relevant functional readouts. Therefore, although these experiments do not capture the full long-term complexity of *in vivo* liver physiology, they provide mechanistically relevant evidence that the engineered device recapitulates key hepatobiliary functions. To achieve a more mature function and expand applications of this device, it is possible to optimize the current design based on the below considerations. This device is a pumpless system, and media in the large reservoirs can maintain cells for 48 h. For longer cell culture, medium replacement is required to further extend the survival of cells. Alternatively, syringe pumps can be further added to prolong the device function and precisely control the fluidic flow to mimic the *in vivo* conditions. The developed methodology of hMSC differentiation into functional liver cells can be further optimized and applied to generate primary liver cells from patients with disease-specific features. Additionally, the liver-on-a-chip devices are made individually and, thus, are difficult to use for high-throughput assays. Despite these limitations, this liver-on-a-chip has many advantages over conventional methods. The *in vivo* physiological relevance and hepatobiliary system of this device are ideal for *in vitro* testing of drug efficacy and toxicity and, potentially, for predictive pharmacokinetics applications.

## Resource availability

### Lead contact

Requests for further information and resources should be directed to and will be fulfilled by the lead contact, Dr. Haolu Wang (h.wang21@uq.edu.au).

### Materials availability

This study did not generate new unique reagents.

### Data and code availability

• RNA sequencing data reported in this paper have been deposited on Zenodo: https://doi.org/10.5281/zenodo.20064005 and are publicly available as of the date of publication.
• This paper does not report original code.
• Any additional information required to reanalyze the data reported in this paper is available from the lead contact upon request.

## Acknowledgments

This work was funded by the 10.13039/501100000925National Health and Medical Research Council of Australia (APP1141121 and APP2008698) and 10.13039/501100001056Gallipoli Medical Research. This research was carried out at the 10.13039/501100020492Translational Research Institute, Woolloongabba, QLD 4102, Australia. The authors acknowledge the Translational Research Institute for providing the excellent research environment and core facilities that enabled this research, particularly the Microscopy Facility. Translational Research Institute is supported by grants from the Australian and Queensland Governments. The ALB-targeting antibody was a gift from Professor Nathan Subramaniam at Queensland University of Technology.

## Author contributions

Conceptualization, H.W., C.-X.Z., and X. Liang; methodology, A.B., H.Y., D.S.B., S.R., D.Z., S.G., K.B., D.H.G.C., X. Liu, H.W., and C.-X.Z.; investigation, A.B., H.Y., D.S.B., S.G., and Q.R.; writing – original draft, A.B. and X. Liang; writing – review & editing, H.W., C.-X.Z., X. Liang and H.Y.; funding acquisition, H.W., C.-X.Z., X. Liang, M.S.R., and D.H.G.C.; resources, M.R.D., H.W., C.-X.Z., and X. Liang; supervision, H.W., C.-X.Z., and X. Liang.

## Declaration of interests

The authors declare no competing interests.

## STAR★METHODS

### Key resources table

**Table:** 

REAGENT or RESOURCE	SOURCE	IDENTIFIER
**Antibodies**		

Mouse anti-CK7 (clone: RCK105)	Abcam	Cat# ab9021; RRID: AB_306947
Rabbit anti-CK19 (clone: EP1580Y)	Abcam	Cat# ab52625; RRID: AB_2281020
Rabbit anti-GPCR TGR5 antibody	Abcam	Cat# ab72608; RRID: AB_2112165
Rabbit anti-β-catenin (clone: E247)	Abcam	Cat# ab32572; RRID: AB_725966
Mouse anti-CFTR (clone: CF3)	Abcam	Cat# ab2784; RRID: AB_303297
Rabbit anti-SOX9 (clone: EPR14335-78)	Abcam	Cat# ab185966; RRID: AB_2728660
Mouse anti-HNF4α (clone: K9218)	Abcam	Cat# ab41898; RRID: AB_732976
Rabbit anti-CXCR4	Abcam	Cat# ab13854; RRID: AB_13854
Mouse anti-FOXA2 (clone: 7E6)	Abcam	Cat# ab60721; RRID: AB_941632
Rabbit anti-CD44	Abcam	Cat# ab157107; RRID: AB_2847859
Rabbit anti-VIM (clone: EPR3776)	Abcam	Cat# ab92547; RRID: AB_10562134
Mouse anti-ALB (clone: ALB/2356)	NeoBiotechnologies	Cat# 213-MSM6; RRID: 213-MSM6
Mouse anti-BSEP (clone: F-6)	Santa Cruz Biotechnology	Cat# sc-74500; RRID: AB_2242103
Mouse anti-α-tubulin (clone: 6-11B-1)	Abcam	Cat# ab24610; RRID: AB_448182

**Chemicals, peptides, and recombinant proteins**		

EGM-2	Lonza	Cat# CC-3162
Human epidermal growth factor (hEGF)	Peprotech	Cat# AF-100-15
Human fibroblast growth factor-basic (bFGF)	Peprotech	Cat# 100-18B
Hepatocyte growth factor (HGF)	Peprotech	Cat# 100-39H
Vascular endothelial growth factor (VEGF)	Gibco	Cat# PHC9391
Human insulin-like growth factor-1 (IGF-1)	Gibco	Cat# PHG0071
Ascorbic acid	Sigma-Aldrich	Cat# A92902
Nicotinamide	Sigma-Aldrich	Cat# N0636
Oncostatin M	Abcam	Cat# ab270077
Insulin	Sigma-Aldrich	Cat# I9278
Hydrocortisone hemisuccinate	Sigma-Aldrich	Cat# H2270
Interleukin-6 (IL-6)	Gibco	Cat# 200-06
ITS+ premix	Corning	Cat# 354352
Sodium taurocholate hydrate	Sigma-Aldrich	Cat# T4009
Dexamethasone	Sigma-Aldrich	Cat# D1756
Sodium butyrate	STEMCELL Technologies	Cat# 72242
Polydimethylsiloxane (PDMS)	Dow	Cat# 04019862
Poly-L-lysine	Sigma-Aldrich	Cat# P4707
Rhodamine 123 (RHD123)	Sigma-Aldrich	Cat# 83702
Cyclosporine A (CyA)	Sigma-Aldrich	Cat# SML1018
Verapamil hydrochloride	Abbott Laboratories	Cat# M075.784
5(6)-carboxy-2′,7′-dichlorofluorescein diacetate (CDFDA)	Sigma-Aldrich	Cat# 21879
Mk571	Sigma-Aldrich	Cat# M7571
Matrigel	Corning	Cat# 354277
Heparin	Hospira	Cat# 16349
SYBR Green Master Mix	Thermofisher	Cat# 4309155
Triton X-100	Sigma-Aldrich	Cat# T8787
Bovine serum albumin (BSA)	Sigma-Aldrich	Cat# A7906
NucBlue™ Live ReadyProbes™ Reagent (Hoechst 33342)	Thermofisher	Cat# R37605
NucBlue™ Fixed ReadyProbes™ Reagent (DAPI)	Thermofisher	Cat# R37606
4 kDa fluorescence isothiocyanate (FITC)-dextran	Sigma-Aldrich	Cat# FD4
20 kDa fluorescence isothiocyanate (FITC)-dextran	Sigma-Aldrich	Cat# FD20
CellTracker™ Red	ThermoFisher	Cat# C34552
Rat tail type I Collagen	Corning	Cat# 354236
Mouse laminin	Gibco	Cat# 23017015

**Critical commercial assays**		

RNeasy Plus Mini Kit	Qiagen	Cat# 74134
RNeasy Plus Micro Kit	Qiagen	Cat# 74034
SensiFast cDNA Synthesis Kit	Bioline	Cat# BIO-65053
Human SDF-1α ELISA kit	Abcam	Cat# ab100637

**Deposited data**		

Bulk RNA sequencing data	This paper	https://doi.org/10.5281/zenodo.20064005

**Experimental models: Cell lines**		

Human mesenchymal stem cells	Doran M. Lab	N/A
Endothelial colony forming cells	Sormani Le Bourhis L. Lab	N/A
Immortalized human hepatocyte	Schippers et al.^55^ Kuipers F. Lab	RRID: CVCL_8278

**Experimental models: Organisms/strains**		

Male Wistar rats	Animal Resource Center	RRID: RGD_13508588

**Oligonucleotides**		

Primers for RT-qPCR	This paper	see Table S1

**Software and algorithms**		

AutoCAD	Autodesk	https://www.autodesk.com/products/autocad/overview
The DermaInspect System	Jen-Lab GmbH	https://jenlab.de/dermainspect/
ImageJ	National Institutes of Health	https://imagej.net/ij/
GraphPad Prism	GraphPad Software	https://www.graphpad.com

**Other**		

8-well on glass detachable well	Sarstedt	Cat# 94.6170.802
Microfluidic device	This paper	N/A

### Experimental model and study participant details

#### Animals

All animal experiments were approved by animal ethics committee at the University of Queensland (PAH/074//10/NHMRC). Male Wistar rats weighing around 300–350 g (10–12 weeks) were used for all the experiments to reduce inter-animal variability and to align with established experimental models of hepatobiliary transport. As the study focused on this defined transporter-based functional readout and not on sex-dependent liver physiology, sex was not included as an experimental variable. Rats were purchased from Animal Resource Center and were housed in the local animal facility for at least one week before procedures. The procedure was as described previously.^56^ All rats were anesthetized by intraperitoneal injection of ketamine (80 mg/kg) and xylazine (10 mg/kg). The jugular vein was cannulated for drug administration. A midline laparotomy was performed, and the left lobe of the liver was exposed for imaging. Animals were kept anesthetized during the whole procedure and body temperature was maintained at 37°C by placing them on a heating pad. One group of rats was injected RHD123 alone via jugular vein at the dose of 2 mg/kg, while CyA (2.5 mg/kg) was administered to the other group of rats via portal vein 10 min before the injection of RDH123. Rat liver images were acquired at 150 min after RHD123 injection.

#### Cell lines and cell culture

All cells were cultured in 37°C and 5% CO_2_. Human MSCs were generously provided by Associate Professor Michael Doran at Queensland University of Technology. Briefly, human MSCs were isolated from bone marrow aspirates collected from three fully informed healthy donors who provided written consent. The healthy volunteer donors are recruited from Mater Private Hospital, Brisbane, Australia. Ethical approval is granted through the Mater Health Services Human Research Ethics Committee and ratified by the Queensland University of Technology Human Ethics Committee (number: 1000000938). hMSCs were cultured and characterized from different individual donors as previously described.^55^ MSCs were cultured in low glucose DMEM with 10% FBS, heparin and FGF-2 supplement until confluent. ECFC cells (endothelial colony forming cells) were acquired from Dr. Laura Sormani Le Bourhis (University of Queensland) and cultured in EGM-2 (Lonza, cat. CC-3162). Immortalized human hepatocyte (IHH) cells^57^ were cultured in high glucose DMEM supplemented with 10% FBS.

#### Cholangiocyte differentiation

Briefly, human MSCs were treated for 2 days with serum deprived media containing Iscove’s Modified Dulbecco’s Media (IMDM) (Life Technologies), 20 ng/mL human epidermal growth factor (hEGF), and 10 ng/mL human fibroblast growth factor-basic (bFGF). Differentiation to hepatoblasts was induced by IMDM supplemented with 20 ng/mL hepatocyte growth factor (HGF), 10 ng/mL bFGF, and 0.61 g/L nicotinamide for 7 days. Cells were then treated with reduced media (William’s E Media, 2% FBS, 1 μg/ml insulin, 1 μm hydrocortisone hemisuccinate) for one day. Cells were cultured for another two days in reduced media supplemented with 10 ng/mL interleukin-6 (IL-6). Sodium taurocholate hydrate (10 nM) was added to reduced medium and cells were cultured for 2 days, then medium was replaced with 10 nM sodium taurocholate and 1.8 μm sodium butyrate in reduced medium and cultured for another 2 days.

#### Hepatocyte differentiation

Human MSCs were differentiated into hepatocytes as previously described.^29^^,^^58^ hMSCs were treated for 2 days with serum deprived media containing IMDM (Life Technologies), 20 ng/mL hEGF, and 10 ng/mL bFGF. Differentiation to hepatoblasts was induced by IMDM supplemented with 20 ng/mL HGF, 10 ng/mL bFGF, and 0.61 g/L nicotinamide for 7 days. Cells were then treated with maturation medium consisting of IMDM supplemented with 20 ng/mL oncostatin M, 1 μmol/L dexamethasone, and 50 mg/mL ITS+ premix thereafter.

#### Endothelial differentiation

Human MSCs were differentiated into endothelial cells following a published protocol.^15^^,^^59^ hMSCs were first cultured for 14 days in endothelial differentiation medium consisting of low glucose DMEM supplemented with 2% FBS, 50 ng/mL vascular endothelial growth factor (VEGF), 10 ng/mL bFGF, 5 ng/mL hEGF, 50 μg/mL ascorbic acid, 20 ng/mL human insulin-like growth factor-1 (IGF-1), and 5 μg/mL heparin. Thereafter, the derived cells were cultured in EGM-2.

### Method details

#### MSC-cholangiocyte organoid generation

At Day 20–23, MSC-derived cholangiocytes were trypsinized with 0.25% trypsin-EDTA (Life Technologies) and 4E4 seeded into 0.8 cm^2^ squared wells of an 8-well on glass detachable wells (Sarstedt, cat. 94.6170.802) in 250 μL Matrigel mixture (DMEM, 0.1% Collagen type I, Matrigel 33%). Once the gel set (approx. 2 h), an additional 250 μL of reduced medium was added. The cysts were observed for up to 10 days, stained for CK7, CK19, CFTR, SOX9, α-tubulin and β-catenin, and tested for P-gp transporter function.

#### Fabrication of microfluidic device

The microfluidic device was designed in AutoCAD. This single-layer device was designed with three parallel microchannels. The outer channels are inverted relative to each other. The channels are separated by a line of short trapezoidal pillars (150 μm × 90 μm x 180 μm) spaced at 70 μm. The horizontal length of the channels is 5.07 mm. The central channel is 600 μm wide and the outer channels are 300 μm. The central channel acted as the Space of Disse and held hepatocytes. One outer channel acted as the biliary channel, and the other was the endovascular channel. The inner channel inlet is 1.5 mm in diameter, and the outlet is 2 mm in diameter. The outlet end of the inner channel was tapered to restrict outflow and confine spheroids. The outer channels’ inlets are 2 mm in diameter, and the outlets are 5.2 mm in diameter. The PDMS chip was hole punched at the inlets and outlets, and bonded to a PDMS-coated glass slide.

The microfluidic device was developed following standard photolithography and soft lithography processes as described previously with some modifications.^60^ PDMS, a silicon-based synthetic polymer, is most commonly used in fabricating microfluidic devices for cell culture due to its low-cost, easy fabrication, non-toxic, porosity, biocompatibility and gas permeability.^61^ The microfluidic device was designed in AutoCAD software. The design was then printed to a Chrome Mask using a direct laser writer (157 nm F2 laser exposure system). To fabricate the wafer, a 250 μm SUEX dry film was laminated onto a clean, HF treated 4-inch silicon wafer and then soft baked on a Digital Programable Hotplate at 65°C for 30 min and cooled until 23°C to remove defects or bubbles acquired during lamination and improve resist adhesion to the wafer. Changes in temperature were done gradually at a fixed ramp rate as per equipment protocol. The wafer was UV exposed through the patterned chrome mask at 1500 mJ/cm^2^. To crosslink the patterned SUEX and form the structures, the wafer then underwent a post exposure bake for 5 min at 65°C, 10 min at 95°C, 2 min at 65°C, then cooled to 23°C. Changes in temperature were done gradually at a fixed ramp rate as per equipment protocol. The wafer was then developed and washed to remove excess photoresist. The wafer was hard baked at 120°C for 60 min and cooled to 20°C following fixed ramp rates to remove remaining solvents and stabilize printed features. The wafer was silanized to prevent PDMS adhering to the master wafer in future experiments. To create the PDMS microfluidic device, a silicon elastomer and curing agent (SYLGARD184, Dow Corning) were mixed at a 10:1 ratio, degassed, poured on to the SUEX patterned silicon wafer and allowed to polymerize at 80°C for 1 h. The cross linked PDMS slab was peeled from the wafer. The devices were cut out and punched to create inlets and outlets. The PDMS devices were then oxygen plasma cleaned and bonded to PDMS-coated slides, and sterilized prior to use.

#### Liver-on-a-chip formation

Microfluidic devices were sterilized by UV exposure and treated with 0.01% poly-L-lysine (PLL) (Sigma-Aldrich, USA; cat. P4707-50mL) for 2 h at 37°C and dried overnight. Then 0.2 mg/mL rat tail type I collagen (Corning cat. 354236) with spheroids was introduced into central channel. Collagen was removed and channels were treated with 100 μg/mL mouse laminin (Sigma-Aldrich) and incubated at 37°C for 2 h. The device was then washed with PBS to remove unbound fibronectin and laminin. To create the cholangiocyte tube, 1.25 × 10^8^ hMSC-cholangiocytes/mL were gently pipetted into the microchannel. The inlets and outlets were then filled completely to avoid air bubbles and media evaporation. The devices were incubated in a humidified chamber at 37°C and 5% CO_2_ for 40 min upside down, then rotated and left to incubate overnight. To create the endovascular tube, 1 × 10^8^ MSC-derived endothelial cells were gently pipetted into the microchannel and the same procedure was followed.

Hepatic spheroids were generated in 96-well plates. The plates were first filled with 2.5% agarose and cooled until set. Then 2,000–5,000 hMSC-derived hepatocytes were seeded per well. The plates were centrifuged at 500 xg for 5 min, then incubated at 37°C and 5% CO_2_ overnight. Spheroids were cultured for 2–3 days then collected for seeding into the microchannel. Spheroids were seeded before the formation of cholangiocyte and sinusoidal endothelial tubes into a collagen gel in the center channel.

#### Quantitative reverse transcriptase PCR (RT-qPCR)

RNA from cells was isolated using QIAGEN RNeasy Plus Mini Kit or RNeasy Plus Micro Kit, and 500ng RNA were used for cDNA synthesis using SensiFast cDNA Synthesis Kit. Quantitative PCR was carried out with SYBR Green Real-Time PCR Master Mix (Thermofisher, Australia) in 384-well plates by the Applied Biosystems ViiA 7 Real-Time PCR System. Relative mRNA expression was calculated using comparative threshold cycle (2^−ΔΔCt^) method and normalized against *RLP13A* expression.

#### RNA sequencing and analysis

The RNA from two biological replicates of hMSCs and hMSC-cholangiocytes which were pooled from three samples individually, was extracted and purified using RNeasy Plus Mini Kit (Qiagen). The extracted RNA was sent to Beijing Genomics Institute (BGI) for quality control, sequencing and bioinformatic analysis. The quality of the extracted RNA was assessed using Agilent 2100 Bioanalyzer. All the samples had an RNA Integrity Number (RIN) > 9 and a 28S/18S > 1. RNA sequencing was performed using the DNBseq platform. Briefly, the poly-A containing mRNA was purified using poly-T oligo-attached magnetic beads. After purification, the fragmentation of mRNA was performed using divalent cations. The first strand cDNA was synthesized from the RNA fragments using reverse transcriptase and random primers. The second strand cDNA was synthesized using DNA Polymerase I and RNase H. The cDNA was then added with a single ‘A' base, followed by subsequent adapter ligation. The cDNA library was purified and enriched by PCR and quantified by Qubit. The final library was constructed by pooling the samples to make a single-strand DNA circle (ssDNA circle). DNA nanoballs (DNBs) were then generated from the ssDNA circle by rolling circle replication (RCR). The DNBs were loaded into the nanoarrays and pair-end reads of 100 bp were generated. The sequencing reads that contained >5% unknown N base or adapters, mapped to rRNA, or with a quality score <15 were filtered and removed. The samples with filtered reads had a read coverage ranging from 48.39 million to 48.6 million. The filtered reads were then mapped to the reference genome using HISAT2.^62^ Transcripts were reconstructed using StringTie,^63^ and 13,622 novel transcripts were identified using Cuffcompare.^64^ These novel transcripts were then merged with reference transcripts and mapped with filtered reads using Bowtie2.^65^ The expression levels of genes were calculated using RSEM,^44^ and the differentially expressed genes (DEGs) were identified using DEseq2.^66^ A curated list of MSC and cholangiocyte markers was acquired from the literature. The heatmap of marker expression in each sample was plotted using ClustVis.^67^

#### Immunofluorescent staining

Antibodies targeting CK7 (ab9021, Abcam), CK19 (ab52625, Abcam), TGR5 (ab72608, Abcam), SOX9 (ab185966, Abcam), HNF4α (ab41898, Abcam), CFTR (ab2784, Abcam), ALB (213-MSM6, NeoBiotechnologies), and BSEP (sc-74500, Santa Cruz Biotechnology) were used for immunofluorescent staining. Cells were stained for various proteins and cytokines following manufacturer’s protocol. For both antibody and SNA staining, cells were first fixed with cold 4% paraformaldehyde (PFA) for 15 min at room temperature. The cells were washed with PBS twice then treated with 0.2% Triton X-100 in PBS for 5 min. Cells were rinsed with PBS three times. The cells were then blocked with 5% BSA in PBS for 30 min at room temperature. The primary or conjugated antibody was diluted (1:100 or as specified) in blocking solution and the cells were left at 4°C overnight. Cells were washed with PBS three times. Cells stained with primary antibody were then incubated with the secondary antibody diluted in blocking solution (1:1000 or as specified) for 1 h at room temperature. Nuclear DNA was stained and mounted with either NucBlue Fixed ReadyProbes (DAPI) or NucBlue Live ReadyProbes Reagent (Hoechst 33342) for 10 mins at room temperature. Images were taken with Olympus FluoView FV1200 Confocal Microscope (CLSM, Zeiss 710, Jena, Germany).

#### Collagen gel preparation

Collagen gel was prepared from stock type I rat tail collagen, 10X PBS, filtered deionized water and 0.5 NaOH. Reagents were kept in an ice bath. Collagen was mixed with the reagents to a pH of 7.4 and a final concentration of 2.5 mg/mL. After mixing well, collagen gel was carefully introduced into the central channel which had been prior coated with poly-L-lysine and dried overnight. The chip was then placed in a humidified Petri dish with excess PBS and placed into an incubator at 37°C for 30 min. To prevent shrinkage, pre-warmed DMEM or coating was introduced into the side channel.

#### Functional assay

The RHD123 transport assay was performed to determine the presence or absence of a lumen in organoids and bile duct channels. Cholangiocyte organoids or bile duct channels were incubated with 100 μM RHD123 (Sigma 83702) for 5 min at 37°C then washed three times with William’s E Medium and incubated for a further 30 min at 37°C. Images were taken using a confocal microscope. To inhibit P-gp transporter, cells were incubated with 10 μM verapamil hydrochloride for 45 min prior to addition of RHD123. The fluorescent intensity of the bile ducts and spheroids with or without verapamil were compared using a corrected total cell fluorescence (CTFC) ratio, where CTFC = Integrated Density – (Area of selected cell × Mean fluorescence of background readings). For evaluation of MRP2 function, 10 μM CDFDA was treated in cells for 20 min at 37°C then washed twice with PBS and incubated for a further 20 min. Images were taken using a confocal microscope. To inhibit MRP2 transporter, cells were incubated with 50 μM MK571 for 20 min prior to addition of CDFDA.

#### Intravital imaging

The DermaInspect System (Jen-Lab GmbH) was used for multiphoton imaging of rat livers. The excitation wavelength was set at 740 nm for liver autofluorescence and at 900 nm for selective detection of RHD123. Fluorescence emission in the spectral range of 350–650 nm was collected by a band-pass filter (BG39). The depth of imaging area was about 30 μm under the liver surface.

#### Dextran gradient test

To ensure collagen gel would sustain a chemokine gradient and to visualize the SDF-1α gradient, 20 μg/mL 4 kDa or 20 kDa fluorescence isothiocyanate (FITC)-dextran (Sigma-Aldrich) was introduced into one of the side channels and imaged immediately. Fluorescence images were taken every 10 s for 5–10 min, and every 10 min up to 1 h. ImageJ (NIH image, Wayne Rasband) was used to measure and plot profile the fluorescence intensity. Maximum dextran fluorescent intensity was measured in five regions of interest (ROIs): the source channel (bile duct channel), the collagen ROI (hepatocyte channel), the target MSC-channel ROI (sinusoidal channel), and two pillar gap ROIs between the channels. The fluorescence in the ‘source’ channel was set to 1.0 and the rest was made relative accordingly.

#### MSC 3D migration assay

For MSC migration assay, hMSCs which were induced into sinusoidal channel were stained with CellTracker Red (ThermoFisher) according to manufacturer’s instructions. Live cell Images for monitoring of hMSC migration were obtained by Olympus IX73 Inverted Microscope every 24 h for 2 days. Migration over 24 h was obtained using the DeltaVision Elite Deconvolution Microscope, with images taken every 30 min. Cells were then tracked manually using ‘Trackmate’ in ImageJ. Calculations, statistical analysis and presentation were done in GraphPad Prism 9.4.1. Cells subjected to hypoxia were cultured in a hypoxia chamber incubator at 37°C in 3% O_2_, 5% CO_2_ and 92% N_2_ for 4 h. Imaging was done immediately after.

#### SDF-1α concentration assay

SDF-1α concentration in cell culture media was measured using human SDF-1α ELISA kit (Abcam, ab100637). The experiment was carried out according to the manufacturer’s protocol. Briefly, serum free samples were collected, supernatant was extracted and incubated in the provided ELISA kit reagents and wells. Samples were read at 450 nm on a microplate reader and analyzed in GraphPad Prism 9.4.1.

### Quantification and statistical analysis

All statistical analyses were performed in GraphPad Prism (version 9.4.1). The statistical tests used and exact value of *n* can be found in the corresponding figure legends. Data is represented as mean ± s.d. unless otherwise specified in the figure legends. Statistical comparison of two groups was determined using unpaired two-tailed t-tests or a Mann-Whitney test, and the test is specified in the figure legends. Statistical comparison of multiple groups was done using either a Kruskal-Wallis with Dunnett’s multiple comparison or one-way ANOVAs with Bonferroni correction, and is specified in the figure legend. A p value <0.05 was used to indicate a statistically significant difference.

## Supplemental information

Document S1. Figures S1–S8 and Table S1

Document S2. Article plus supplemental information
