# Supplementary material for: Mesenchymal stem cell-derived liver-on-a-chip reconstitutes human liver structure and function
Source: Cell Rep Methods. 2026 Aug 10;6(10):101545. doi: 10.1016/j.crmeth.2026.101545 (PMC13721747; doi:10.1016/j.crmeth.2026.101545)
Supplement: Supplementary file 1 [file mmc1.pdf]

Cell Reports Methods, Volume *6*

Supplemental information

Mesenchymal stem cell-derived liver-on-a-chip reconstitutes human liver structure and function

Anastasia Brooks, Haotian Yang, Deborah S. Barkauskas, Supun Ranaweera, Da Zou, Sally Goh, Qi Ruan, Kim Bridle, Darrell H.G. Crawford, Xin Liu, Michael S. Roberts, Michael R. Doran, Xiaowen Liang, Chun-Xia Zhao, and Haolu Wang

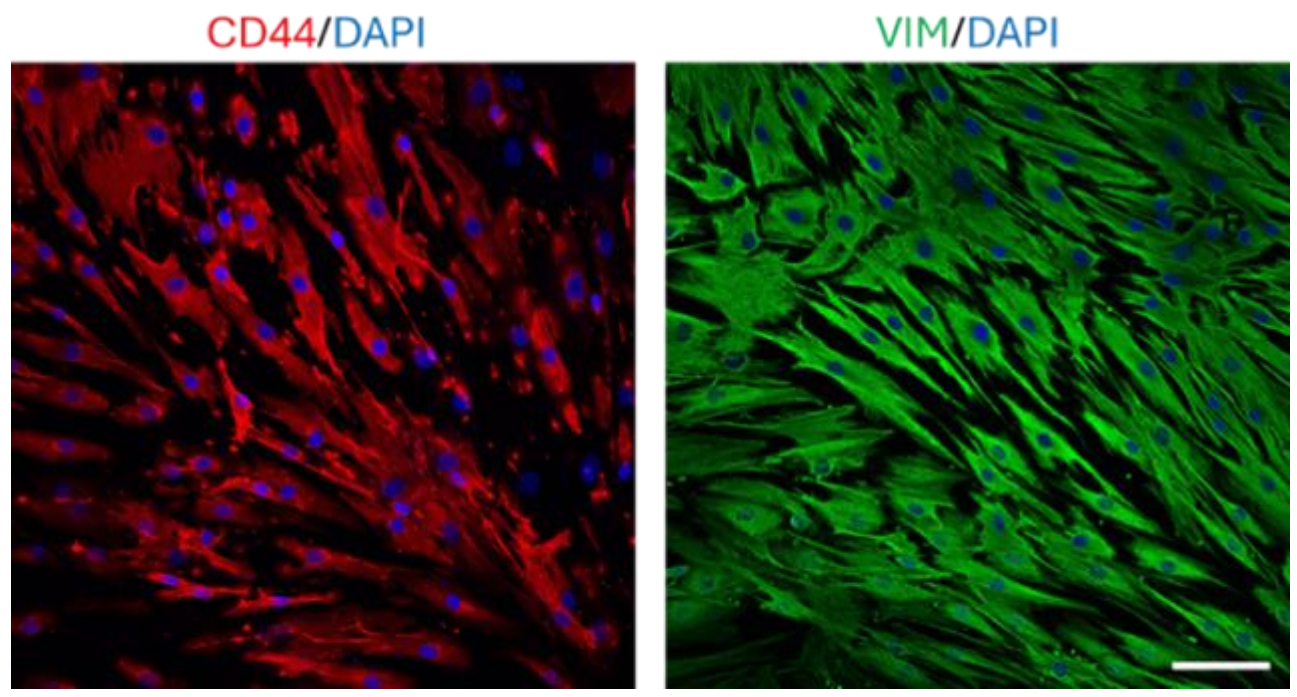

Figure S1. Representative fluorescent images of MSCs stained with CD44 (red), vimentin (VIM, green) and DAPI (blue), related to Figure 1. Scale bar, 100 μm.

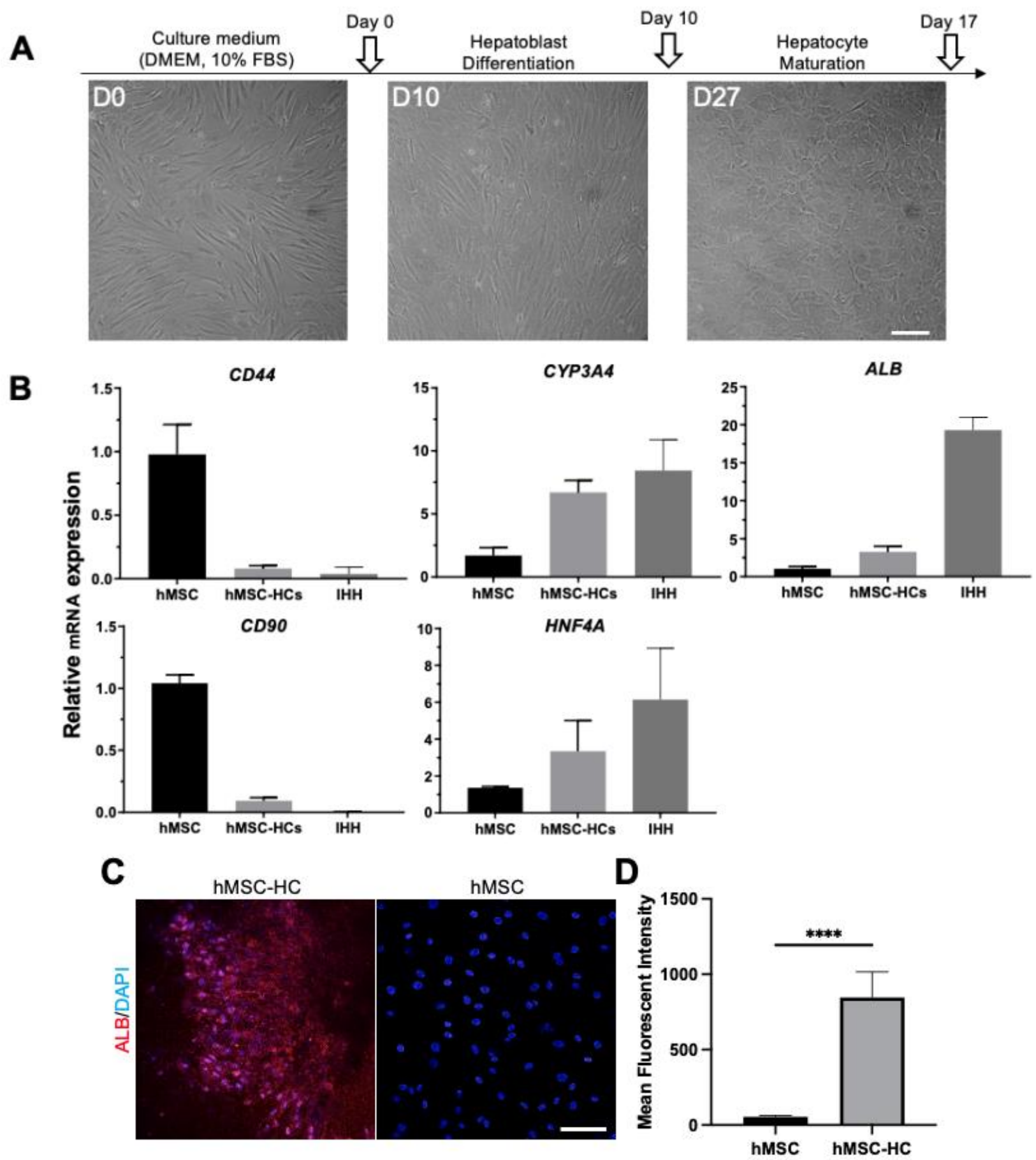

Figure S2. Differentiation of hMSCs into hepatocytes, related to Figure 1.
(A) Schematic timeline of hepatocyte differentiation. Scale bar, 200 µm.
(B) Respective qPCR analyses of gene expression profile of hepatic and stem cell markers in hMSCs, hMSC-derived hepatocytes (hMSC-HCs) and human hepatocytes (IHH, positive control) (n = 3).
(C) Immunofluorescence showing the expression of hepatocyte marker ALB in the spheroid formed by hMSC-HCs and in hMSCs. Scale bar, 100 µm.
(D) Quantification of ALB immunofluorescence in individual cells of hMSC-HCs and hMSCs (n = 30). Significance was determined using unpaired t-test. ****, $p<0.0001$.
All error bars indicate ± s.d.

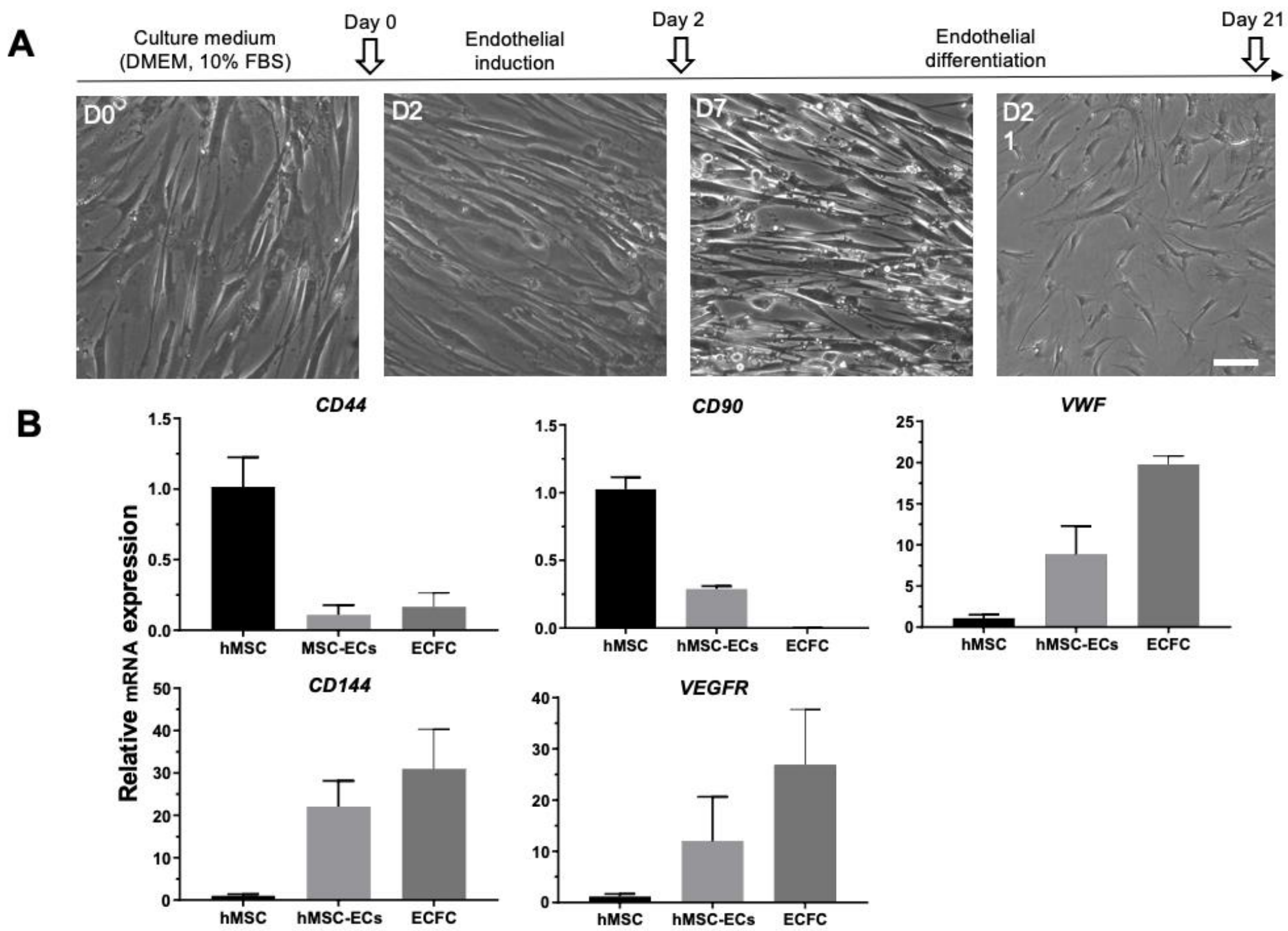

Figure S3. Differentiation of hMSCs into endothelial cells, related to Figure 1.
(A) Schematic timeline of endothelial cell differentiation. Scale bar, 100 µm.
(B) Respective qPCR analyses of gene expression profile of endothelial and stem cell markers in hMSCs, hMSC-derived endothelial cells (hMSC-ECs) and Endothelial colony forming cells (ECFCs, positive control) (n = 3). All error bars indicated ± s.d.

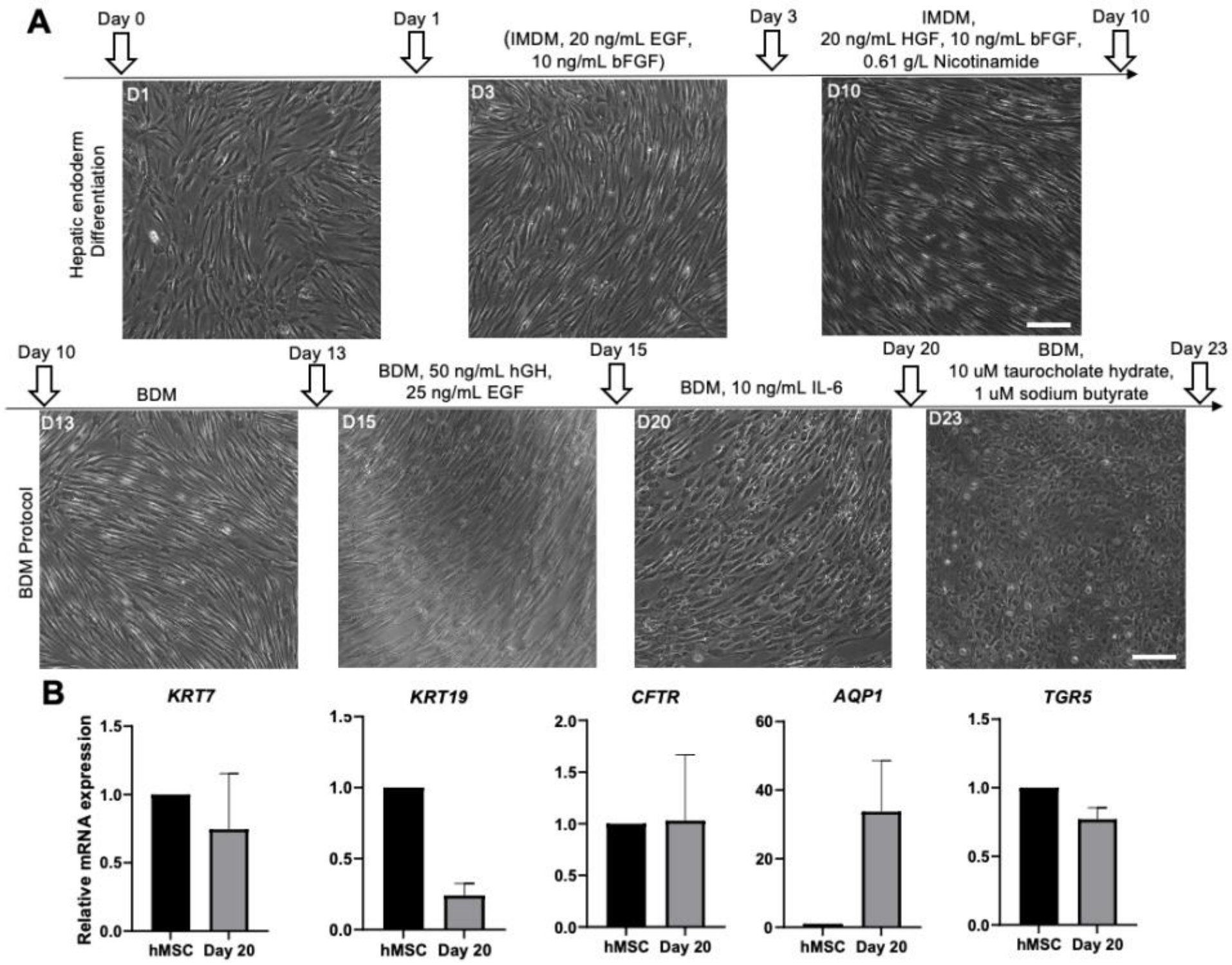

Figure S4. Differentiation of hMSC into cholangiocytes using BDM protocol, related to Figure 1.
(A) Schematic timeline of cholangiocyte differentiation using BDM protocol. Scale bar, 200 µm.
(B) Respective qPCR analyses of gene expression profile of cholangiocyte markers using BDM protocol. Error bars indicated ± s.d.

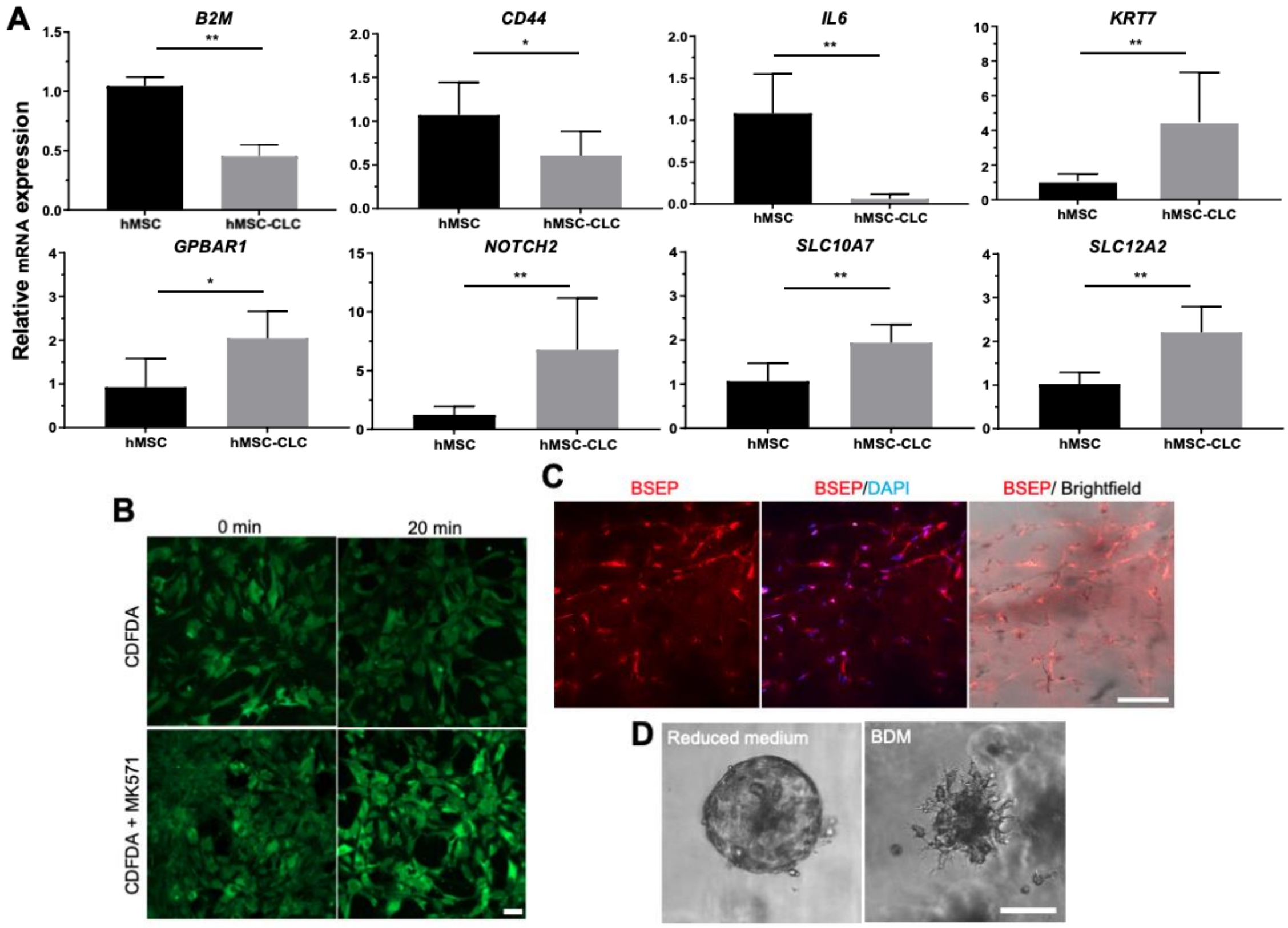

Figure S5. Characterization of hMSC-cholangiocytes (hMSC-CLCs), related to Figures 1 and 2.
(A) RNA sequencing data was confirmed by qPCR analyses for key genes (n = 3). Error bars indicated ± s.d. Significance was determined using unpaired t-test. *p<0.05; **p<0.01.
(B) Fluorescent imaging showing the efflux of CDF via MRP2 and the inhibition by MK571 in hMSC-cholangiocyte.
(C) Immunofluorescence showing the expression of BSEP in the tubular structure formed by hMSC-CLCs.
(D) Organoid formation of hMSC-derived cholangiocyte-like cells generated from two protocols: reduced medium and BDM.
Scale bar, 100 µm.

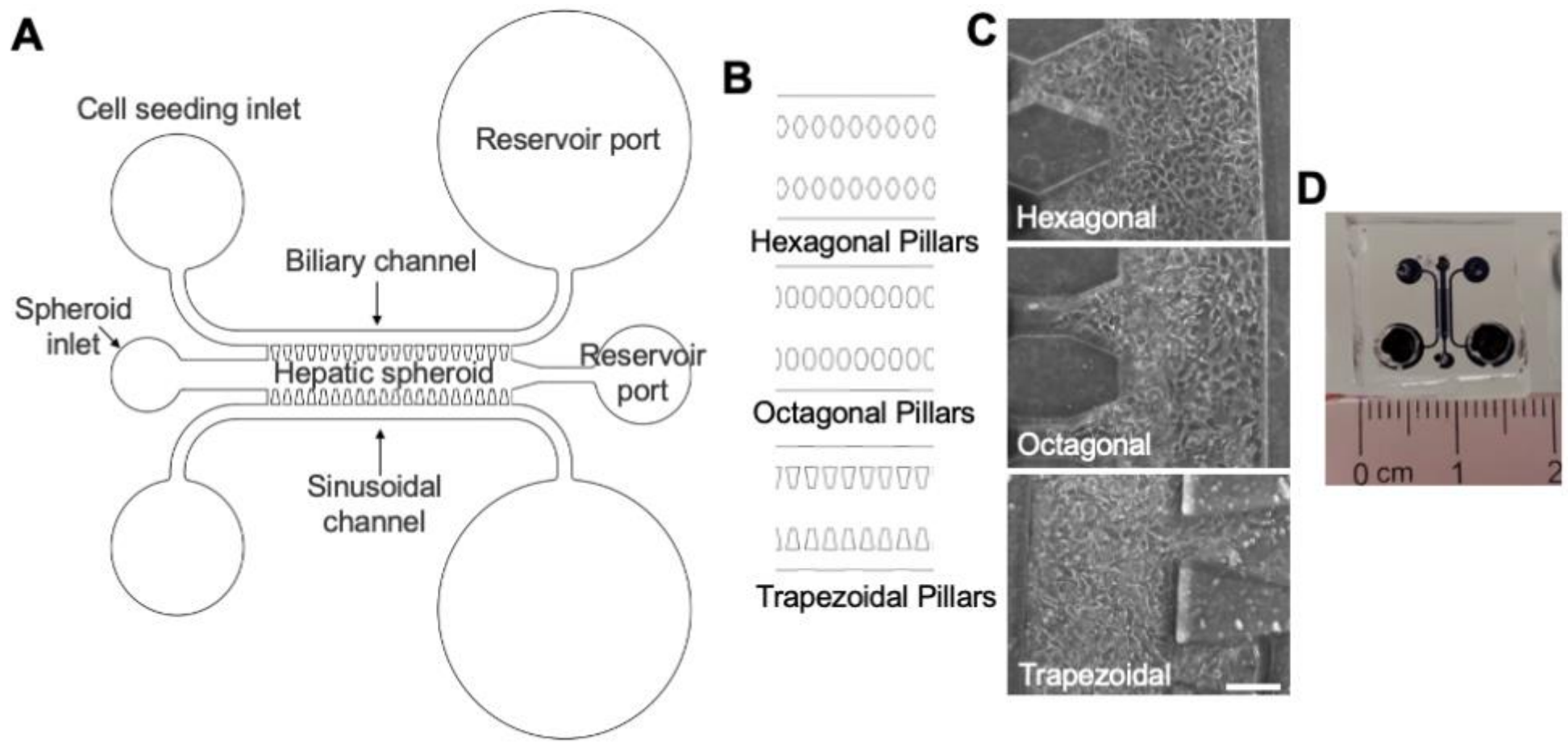

Figure S6. Characterization of the second device with larger outlets, related to Figure 3.
(A and B) Design and fabrication of the second device with larger outlets (A) and different pillar systems (B).
(C) Bright field images of CLC infiltrated into the gap of pillars. Scale bar, 100 µm.
(D) Photograph of the second liver-on-a-chip device filled with dyes.

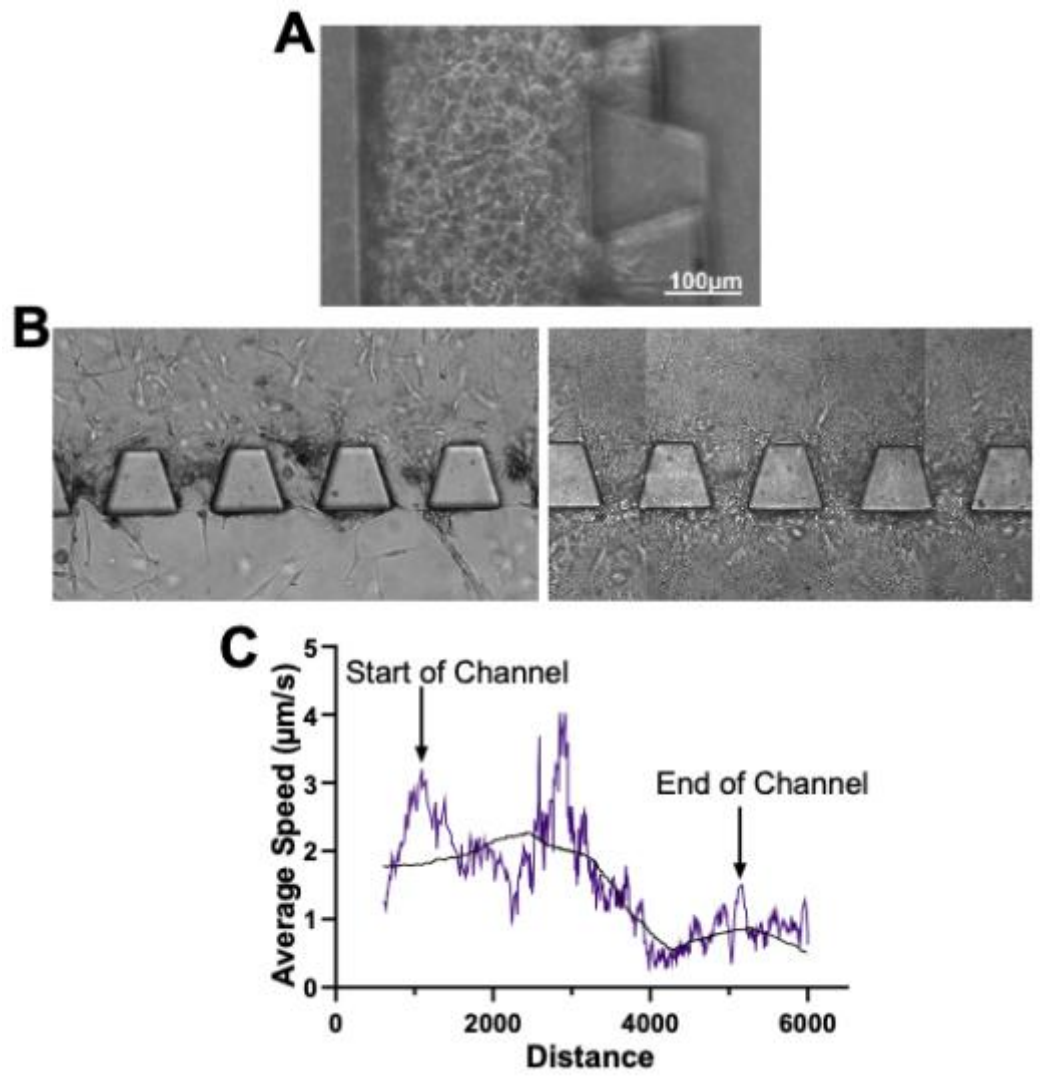

Figure S7. Characterization of the hMSC-derived liver-on-a-chip microfluidic, related to Figure 3.
(A) Bright field image showing hMSC-ECs were seeded onto the barrier between channels at confluent density.
(B) Bright field images showing SDF-1-induced cell migration through the channels without endothelial coating (Left) or when endothelial cells were present (Right).
(C) Representative tracing of NPs flowing through the lumen. Smoothed data of the average speed of DiI-loaded NPs. Smoothing was done by the method of Savistsky and Golay to highlight the overall trend. The average flow rate was calculated to be 0.04 µl/min in empty channels, and 0.006 µl/min in channels with bile ducts and vasculature.

Time (min)	Left end	Middle	Right end
60	1.90E+07	2.40E+07	2.08E+07
120	1.97E+07	1.91E+07	1.81E+07

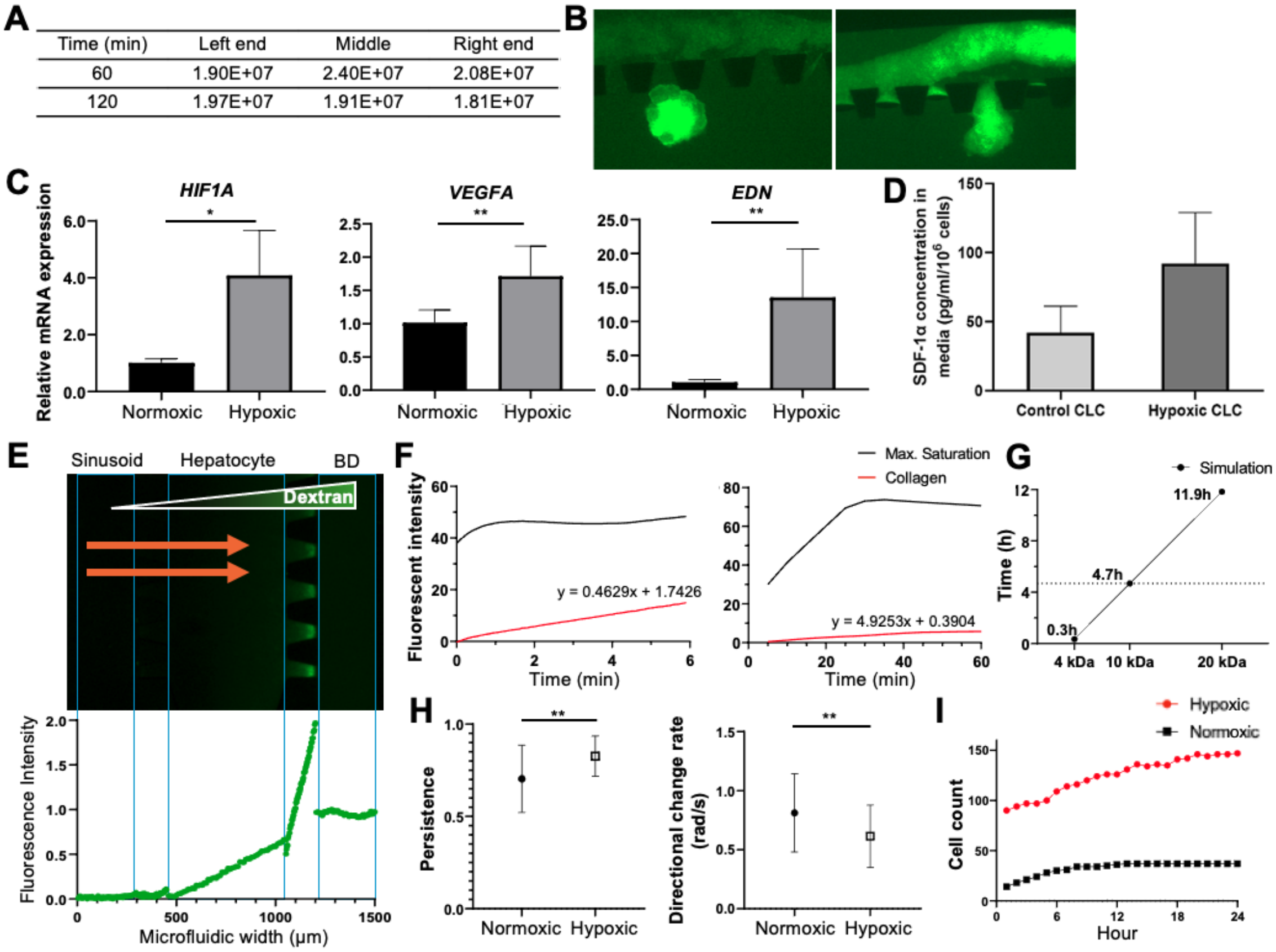

Figure S8. Functional properties of hMSC-derived liver-on-a-chip, related to Figure 4.
(A) Table showing the fluorescent intensity of RHD123 in the representative hMSC-HC spheroids at different positions of the channel.
(B) Representative fluorescent images of RHD123 in hMSC-HC spheroids at the left (Left) and right (Right) ends of the channel.
(C) qPCR analyses of hypoxic markers in hMSC-CLCs including *HIF-1α*, *VEGFA*, and *EDN* (n = 3). Significance was determined using unpaired t-test.
(D) SDF-1α concentration was quantified in control and hypoxic hMSC-CLCs in microfluidic (n = 2).
(E) A FITC-dextran gradient was established in the collagen gel matrix in the central channel to mimic SDF-1α gradient. Blue squares depict borders between channels. The orange arrows indicate the direction of MSC migration.
(F) Plot profile of 4 kDa (Left) or 20 kDa (Right) dextran saturation throughout the region of interest versus maximum measured intensity over 6 mins.
(G) Estimated time taken for saturation for a 10 kDa molecule based on 4 kDa and 20 kDa generated equations.
(H) Persistence index (ratio of cell displacement to total track length) (Left) and mean directional change (ratio of angle between two links to all links) (Right) of MSCs in normoxic (n = 37) or hypoxic (n = 150) conditions. Significance was determined using Mann Whitney test.
(I) Migrating cell counts per hour over 24 hrs in normoxic and hypoxic conditions.
*, $p<0.05$; **, $p<0.01$. Error bars indicated ± s.d.

Table S1. Primer sequences used for RT-qPCR, related to STAR Methods.

Gene	**Forward (5'-3')**	**Reverse (5'-3')**
GATA4	CCAAACCAGAAAACGGAAGC	TGTGCCCGTAGTGAGATGACA
FOXA2	GGAGCGGTGAAGATGGAAGG	CGGCGTTCATGTTGCTCAC
SOX9	GTACCCGCACTTGCACAAC	TCTCGCTCTCGTTCAGAAGTC
AQP1	GTCCAGGACAACGTGAAG	GAGGAGGTGATGCCTGAGAG
CFTR	GCTTCCTATGACCCGGATAAC	GGAGCAGTGTCCTCACAATAA
CD44	CGGACACCATGGACAAGTTT	GAAAGCCTTGCAGAGGTCAG
CYP3A4	CCTTACATATACACACCCTTTGGAAG	GGTTGAAGAAGTCCTCCTAAGCT
ALB	GATGAGATGCCTGCTGACTTGC	CACGACAGAGTAATCAGGATGCC
CD90	AGGACGAGGGCACCTACAC	GCCCTCACACTTGACCAGTT
HNF4A	ACTACATCAACGACCGCCAGT	ATCTGCTCGATCATCTGCCAG
VWF	GACCTTGCTGAAGAGTCATCG	GCCAGTCAGCTTGAAATTCTG
CD144	CCTGTGTTCACGCATCGGTT	GTCTGCATCCACTGCTGTCA
KRT7	CAGGATGTGGTGGAGGACTT	TGAGGGTCCTGAGGAAGTTG
KRT19	CGTCGTCCTTCGGAGGCCT	GGTCAGGACGCCGCCGTAGCCG
TGR5	CTGGCCCTGGCAAGCCTCAT	CTGCCATGTAGCGCTCCCCGT
B2M	CTCCGTGGCCTTAGCTGTG	TTTGGAGTACGCTGGATAGCCT
IL6	CCCTGAAGTACCCGATAGAACA	GGCAACACGAAGCTCATTG
GPBAR1	CAGCCAGGACACCAGACAT	CTCCACACTAGCAACAGGGTC
NOTCH2	TACAGTTGTCGCTGCTTGCC	GACGAAGGTTTCACAGTGCC
SLC10A7	ATAGGGGTGAATGGGGGACC	ACCAAAGCACTGGTCAGCTC
SLC12A2	TGGCACCAAGGATGTGGTAGTA	TCTTGCCATCCTCTTCCTCAAC
HIF1A	TGCTCATCAGTTGCCACTTC	TCCTCACACGCAAATAGCTG
VEGFA	GGAGGAGGGCAGAATCATCAC	CTTGGTGAGGTTTGATCCGC
EDN	CCAGAAACAGCAGTCTTAGGC	TCACGGTCTGTTGCCTTTGT
RLP13A	GTTCCTGCTGCCCTCAAG	GTCACTGCCTGGTACTTCC
